# Beyond the Administrative Label: Municipal Shelter Conversion and Dog Outcomes in Korea, 2016–2025

**DOI:** 10.3390/ani16162505

**Published:** 2026-08-12

**Authors:** Byeong-Cheol Song

**Affiliations:** Agriculture and Livestock Division, Yeongyang County Office, 37 Guncheong-gil, Yeongyang-eup, Yeongyang-gun 36537, Gyeongsangbuk-do, Republic of Korea; sbc07016@korea.kr; Tel.: +82-54-680-6660

**Keywords:** animal welfare, animal shelters, competing risks, dogs, implementation science, municipal governance, shelter management, veterinary public health

## Abstract

Municipal conversion of dog shelters from contracted to direct operation often coincides with changes in facilities, staffing, veterinary access, adoption practices, euthanasia policy, and record keeping. The primary objective was to evaluate associations with municipality-level disposition and throughput outcomes; a secondary exploratory objective examined dog-level adoption and euthanasia pathways. We analyzed 68,032 dogs from 22 municipalities in Gyeongsangbuk-do, Korea, during 2016–2025. The primary difference-in-differences comparison included four municipalities that converted around 2021 and nine contracted controls. Event-study, exact label-permutation, and comparison-pool analyses served as diagnostics. Estimates were imprecise, no exact permutation *p*-value was below 0.05, and adoption and euthanasia had different pre-conversion trends. Excluding five newly digitized controls changed the adoption estimate from +4.65 to −1.86 percentage points. Censor-aware analysis estimated 13.33 additional days without a recorded disposition during the first year, but uncertainty was wide and the day-365 unresolved difference was small. In the secondary 35,366-dog subframe, mixed/Jindo-type outdoor dogs had a lower adoption hazard ratio (HR, 0.39; 95% CI, 0.35–0.45) and a higher euthanasia HR (1.61; 95% CI, 1.37–1.90). Direct operation was not supported as a uniform causal intervention; municipalities should evaluate welfare and management components and target support to dogs at elevated risk.

## Simple Summary

The primary aim of this study was to examine whether municipal conversion of dog shelters from contracted to direct operation was followed by changes in adoption, euthanasia, return-to-owner, and shelter-throughput outcomes. We analyzed 68,032 dogs from 22 municipalities in Korea during 2016–2025. The primary comparison involved four municipalities that converted around 2021 and nine that remained contracted. Event-study and sensitivity analyses showed that the groups already followed different adoption and euthanasia trajectories before conversion and that estimates changed with the comparison pool. Therefore, the data did not support a causal or uniform effect of direct operation. A secondary dog-level analysis found that mixed/Jindo-type outdoor dogs were adopted less often and euthanized more often than small or recognized companion breeds. In practice, municipal reform should be evaluated through measurable operational components—such as veterinary access, housing capacity, adoption and foster support, long-stay review, euthanasia decision processes, and record completeness—rather than the administrative label alone.

## 1. Introduction

Municipal shelters for lost, abandoned, and stray dogs sit at the intersection of veterinary public health, animal welfare, and local administration. They manage a range of shelter processes and outcomes, including return-to-owner, adoption, transfer, euthanasia, recorded death in care, and long-term sheltering. In Korea, national welfare reports, the Animal Protection Management System, and animal protection legislation provide administrative information on rescued animals, facilities, and animal registration, making municipal sheltering an important policy and public health setting [1,2,3,4].

A governance label, however, is not a well-defined intervention. Conversion from contracted to direct operation may coincide with shelter construction, staffing changes, veterinary care arrangements, intake screening, adoption promotion, euthanasia policy changes, and revised record-keeping processes. Studies on shelter standards and prior shelter outcomes show that housing, veterinary care, population management, adoption practices, and organizational capacity shape shelter outcomes [5,6,7,8,9,10,11,12,13,14,15,16,17,18,19,20,21]. Implementation science further distinguishes an intervention label from the content, context, and fidelity of implementation [22]. This distinction is central here because municipalities may adopt the same administrative label while implementing materially different operational changes.

Dog-level case-mix and administrative measurement add further heterogeneity. Mixed/Jindo-type outdoor dogs are common in Korean rural and urban–rural shelters, and phenotype, age, sex, and intake health status can affect adoption, return-to-owner, length of stay, and euthanasia outcomes [9,12,15,16,17,18,19]. Municipal ledgers can also differ in how they record unresolved cases, transfers, deaths, and disposition dates. Analyses that ignore either case-mix or recording practice may overattribute observed differences to governance.

Using a 22-municipality, dog-only administrative census, the primary research question was whether conversion from contracted to direct municipal operation was associated with changes in municipality-level adoption, euthanasia, return-to-owner, recorded death-in-care, and shelter-throughput outcomes. The municipality–year difference-in-differences (DID) analysis addressed this question, while event-study, exact label-permutation, and data-provenance analyses were used only to evaluate the credibility and robustness of that primary comparison. A secondary, exploratory question was whether dog phenotype, age, sex, and intake health status were associated with adoption and euthanasia pathways in a harmonized dog-level subframe. This hierarchy separates the policy comparison from the dog-level welfare analysis and avoids treating statistical diagnostics as independent scientific objectives.

## 2. Materials and Methods

### 2.1. Study Objectives and Analytic Hierarchy

**Figure 1:**
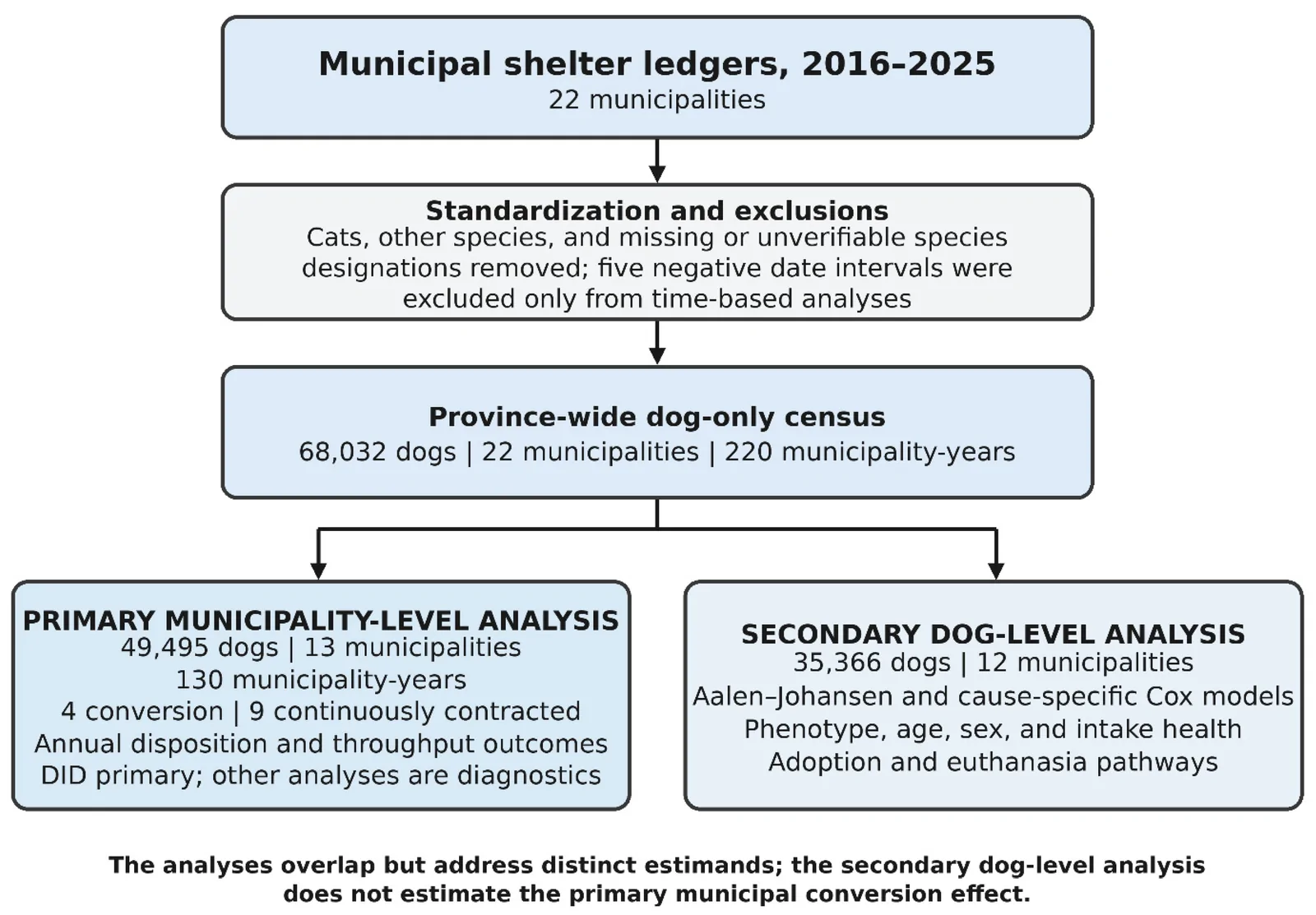
Study design, data cleaning, and analytic hierarchy. Records of cats and other species and those with missing or unverifiable species designations were excluded from analytic denominators. Five records with disposition dates preceding rescue dates were retained in disposition-rate denominators but excluded from length-of-stay and time-to-event analyses. The municipality–year panel is the primary analysis; event-study, permutation, and provenance analyses are diagnostics of the same municipal estimand. The dog-level survival subframe is secondary and addresses adoption and euthanasia pathways. The two frames overlap and do not represent a sequential attrition flow.

Figure 1 summarizes data cleaning and the analytic hierarchy. Excluding records on cats and other species and those with missing or unverifiable species designations, the province-wide census contained 68,032 dogs from 22 municipalities. The primary municipality–year panel included 49,495 dogs from 13 municipalities and evaluated the association between conversion and annual municipal outcomes. Event-study, exact permutation, and control-pool analyses served as diagnostic and sensitivity analyses of this same estimand. The secondary harmonized survival subframe included 35,366 dogs from 12 municipalities and examined dog-level adoption and euthanasia pathways. The primary and secondary frames overlap but address distinct estimands and do not represent sequential stages of attrition.

### 2.2. Study Design, Data Sources, and Analytic Scope

This study covers Gyeongsangbuk-do, Korea, during the period 2016–2025. Gunwi County was excluded because it was incorporated into Daegu Metropolitan City in July 2023. The final dog-only census included 68,032 records from 22 municipalities and 220 municipality–year observations. Seven ledgers that were newly digitized during revision contributed 25,464 records (37.4% of the census). Municipality-level source coverage and provenance are reported in Supplementary Table S1. Reporting followed the STROBE-Vet guidelines [23], and jurisdictional coverage was cross-checked against Korean Statistical Information Service data [24].

Municipalities were grouped as six continuously direct descriptive references, nine continuously contracted controls, four primary 2021-boundary conversions, one independent sensitivity case, and two late-conversion contextual cases. Table 1 summarizes group-level contributions. Municipality names, implementation histories, data provenance, and analytic roles are provided in Supplementary Table S1.

The primary municipality–year DID analysis compared the four converted municipalities with the nine continuously contracted controls and included 49,495 dogs in 130 municipality–year observations. Continuously direct-operation municipalities were used as descriptive references because they experienced no governance transition. The expanded control pool reduced dependence on any single contracted municipality and better represented contemporaneous provincial trends.

The harmonized survival subframe included 35,366 dogs from 12 municipalities: four continuously direct, four continuously contracted, and four converted municipalities. These municipalities had comparable rescue and disposition dates, event definitions, and dog-level covariates. The subframe was used only for the secondary analysis of adoption and euthanasia pathways and not to estimate the primary municipal conversion effect. Municipality membership is listed in Supplementary Table S1.

### 2.3. Dog-Level Variables and Recoding

Municipal records were standardized using rescue date, disposition date, species, breed, age descriptor, sex, intake health status, and final disposition. Eligible records had a rescue date from 1 January 2016 to 31 December 2025 and a verified species designation of dog. Final dispositions were recoded as adoption, return-to-owner, euthanasia, recorded death in care, other/transfer, unresolved/in care, or unresolved/unknown. The source term commonly translated as “natural mortality” was defined operationally as a death recorded while the dog was in shelter care; it did not imply death from old age or a verified natural cause. Length of stay was calculated from rescue to disposition only when a disposition date was recorded.

Unresolved/in-care dogs were retained in the disposition denominator and right-censored on 31 December 2025 for time-to-event follow-up. Accordingly, the mean length of stay and unresolved/in-care share were treated as linked throughput indicators: the former describes dogs with an observed disposition, whereas the latter captures dogs still pending follow-up by the study censoring date. Breed phenotype was recorded as mixed/Jindo-type outdoor dog, small or recognized companion breed, or unknown. Age was categorized as puppy/juvenile (<1 year), adult (1–6 years), senior (≥7 years), or explicitly unspecified; residual age descriptors that could not be classified were retained as missing/unclassifiable for descriptive tabulation. Sex and intake health status were harmonized similarly. Neuter and microchip statuses were not analyzed because recording was inconsistent.

### 2.4. Governance and Implementation Classification

Municipalities were classified a priori according to governance history and transition timing (Table 1; Supplementary Table S1). Direct operation was treated as a heterogeneous implementation package that could include facility construction, staffing, veterinary access, intake screening, euthanasia policy, adoption promotion, and changes in record keeping. Conversion was not randomized, and the content and timing of implementation differed across municipalities. The independent and late-conversion cases were therefore not pooled into the primary DID estimand.

### 2.5. Outcomes

Primary annual municipality-level outcomes included the return-to-owner rate, adoption rate, recorded death-in-care rate, euthanasia rate, complete-case mean length of stay (LOS), and unresolved/in-care or unknown share. Primary censor-aware throughput outcomes were RMST365, defined as the mean of min (time to recorded disposition, 365 days), and the probability of no recorded disposition by day 365. Annual case-mix descriptors included the mixed/Jindo-type outdoor-dog share, puppy/juvenile share, female share, and clinically compromised/injured share. Secondary dog-level outcomes were time to adoption and time to euthanasia, with other dispositions treated as competing events.

### 2.6. Statistical Analysis

#### 2.6.1. Primary Municipality-Level Analysis

Descriptive statistics were calculated for the complete census and analytic groups. The primary municipality–year DID models compared four converted municipalities with nine continuously contracted municipalities, using 2016–2020 as the pre-period and 2021–2025 as the post-period, with municipality and year fixed effects. Because only four municipalities converted, inference emphasized CR1 municipality-clustered standard errors with a t reference and exact two-sided municipality-label permutation *p*-values across all C(13, 4) = 715 assignments. These *p*-values were interpreted as robustness diagnostics rather than design-based randomization probabilities because conversion was observational [25,26,27,28].

#### 2.6.2. Diagnostic and Sensitivity Analyses

Event-study models replaced the single DID term with conversion-status-by-intake-year interactions, using 2020 as the omitted reference. Diagnostics included municipality-clustered confidence intervals, coefficient-specific exact label-permutation *p*-values, an omnibus exact test of the four pre-2020 leads, and a complementary 2016–2020 differential linear pre-trend test. A provenance sensitivity analysis repeated the DID models after excluding five newly digitized contracted controls. For censor-aware throughput, 2016–2024 intake cohorts were used so that each dog could contribute up to 365 days of follow-up; dogs with no recorded disposition or a disposition after day 365 contributed 365 days to RMST365. Detailed HC1 comparisons, complete event-study coefficients, exact permutation diagnostics, control-pool sensitivity estimates, censor-aware summaries, and the exploratory resolution-time Cox model are provided in Supplementary Tables S3–S8 and Supplementary File S1 (Analysis_Workbook.xlsx), thereby keeping the primary presentation focused.

#### 2.6.3. Secondary Dog-Level Analysis

In the 35,366-dog harmonized subframe, nonparametric cumulative incidence functions were estimated with the Aalen–Johansen estimator, using shelter intake as time zero. Cause-specific Cox models were fitted separately for adoption and euthanasia; competing dispositions were censored at their recorded event dates and unresolved dogs were administratively censored on 31 December 2025. Models included phenotype, age, sex, intake health status, municipality fixed effects, and intake-year fixed effects, with municipality-clustered standard errors [29,30,31,32,33]. Because proportional hazards may not hold uniformly and the subframe covered 12 municipalities, estimates were interpreted as descriptive secondary associations rather than municipality-level conversion effects. Analyses were conducted in Python (version 3.13.5) using pandas (version 2.2.3), NumPy (version 2.3.5), statsmodels (version 0.14.6), matplotlib (version 3.10.8), and openpyxl (version 3.1.5).

### 2.7. Ethics, Administrative Authorization, and Data Access

In this study, we used de-identified retrospective administrative records from municipal animal shelters. Official administrative permission and cooperation for academic access and analysis were obtained from the relevant municipalities before the records were used. Each municipality retained authority over its own ledger and over any further disclosure of row-level records. The analytic files contained no owner names, contact information, exact rescue-location identifiers, or other directly identifiable personal information. This study did not involve prospective animal intervention, experimental procedures, or investigator-performed euthanasia. Euthanasia was analyzed only as an administrative final-disposition category recorded in shelter ledgers. Therefore, committee approval for institutional animal care and use was not required.

### 2.8. Use of Generative Artificial Intelligence

During manuscript preparation and revision, ChatGPT (OpenAI; GPT-5.5 Pro and GPT-5.6 Pro; accessed on 9 July, 23 July, and 6 August 2026) was used as an editorial aid for English language editing, manuscript organization, formatting, internal consistency checks, and refinement of the presentation of tables and figures. The AI tool was not used to collect or generate study data, alter the municipal ledgers, select or execute statistical analyses, or determine scientific conclusions. All AI-assisted output was reviewed, verified, and edited by the author, who takes full responsibility for the manuscript.

## 3. Results

### 3.1. Data Provenance, Analytic Denominators, and Implementation Context

The census comprised 68,032 dogs and 220 municipality–year observations. Continuously direct-operation municipalities contributed 11,335 dogs, contracted municipalities 33,871, converted municipalities 15,624, and context/sensitivity cases 7202 (Table 1). Seven newly digitized ledgers contributed 25,464 records (37.4%); five of those ledgers contributed 22,393 control dogs to the primary DID analysis. Thus, the primary municipality-level panel contained 49,495 dogs, and the secondary harmonized survival subframe contained 35,366 dogs. Five negative rescue-to-disposition intervals were excluded from time-based analyses but retained in disposition-rate denominators.

The municipality–year panel and dog-level survival subframe overlap but address distinct estimands and are not sequential stages of attrition (Figure 1). Results are therefore presented in analytic order: the primary municipal conversion analysis first, followed by its diagnostics and throughput analyses, and then the secondary dog-level analysis.

### 3.2. Dog-Level Case-Mix in the Province-Wide Census

Mixed/Jindo-type outdoor dogs represented 81.6% of all admissions, including 86.8% in continuously direct-operation, 79.6% in contracted, and 87.3% in converted municipalities (Table 2). Puppy/juvenile dogs represented 48.5% of admissions, females 51.5%, and clinically compromised/injured dogs 9.6%. Rare, unknown, and unclassifiable values are summarized in the footnote of Table 2 rather than displayed as separate rows.

### 3.3. Crude Group Outcomes

Crude outcomes calculated directly from the municipal ledgers varied substantially among analytic groups before adjustment (Table 3). Contracted municipalities had the highest adoption proportion, whereas converted municipalities had a higher euthanasia proportion and a shorter complete-case mean LOS. Recorded death in care was highest in the continuously direct-operation group. These differences combine operational practice, case-mix, unresolved-record conventions, and data coding and should not be interpreted as unadjusted governance effects.

Supplementary Figure S1 shows crude disposition distributions, Supplementary Figure S2 shows annual intake volume by analytic group, and Supplementary Table S10 reports overall municipality-specific and common-calendar pre/post-outcomes.

### 3.4. Primary Municipality-Level Conversion Analysis

In the primary 13-municipality DID analysis, conversion was associated with −2.19 percentage points in return-to-owner, +4.65 percentage points in adoption, +2.36 percentage points in recorded death in care, −1.80 percentage points in euthanasia, +64.93 days in complete-case mean LOS, and −2.57 percentage points in unresolved/in-care or unknown records (Table 4). Municipality-clustered confidence intervals were wide, and none of the exact studentized permutation *p*-values was below 0.05. The primary analysis therefore did not identify a stable municipality-level change distinguishable from between-municipality variation. Exact label permutation was used as a small-cluster sensitivity diagnostic, not as a randomized causal test, because conversion status was observational.

### 3.5. Diagnostic and Control-Pool Sensitivity Analyses

**Figure 2:**
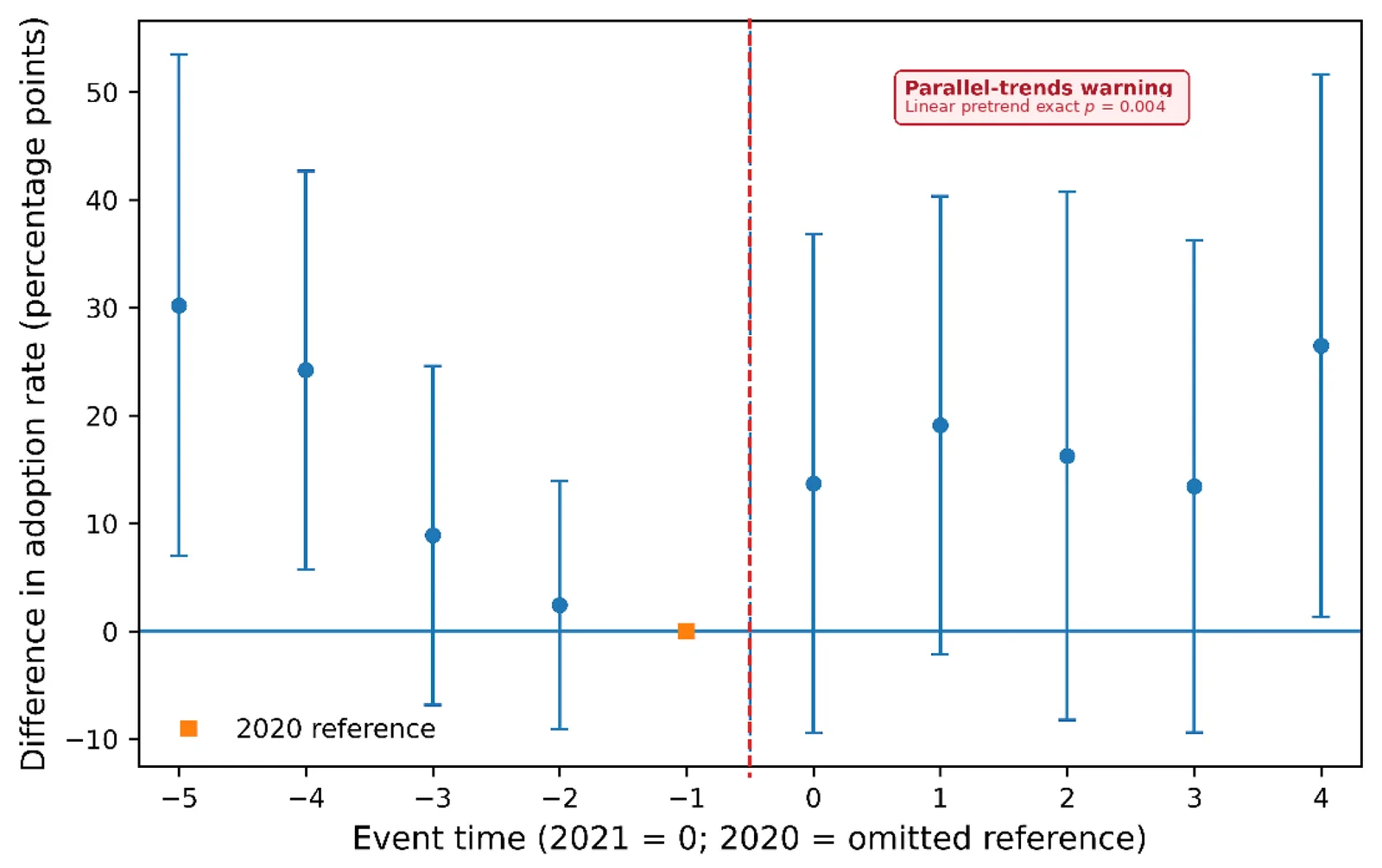
Event-study estimates for adoption rate. The 2020 coefficient is the omitted reference. Points show conversion-minus-contracted differences relative to 2020, and error bars show municipality-clustered t-based 95% confidence intervals. The dashed line separates the pre- and post-2021 periods.

**Figure 3:**
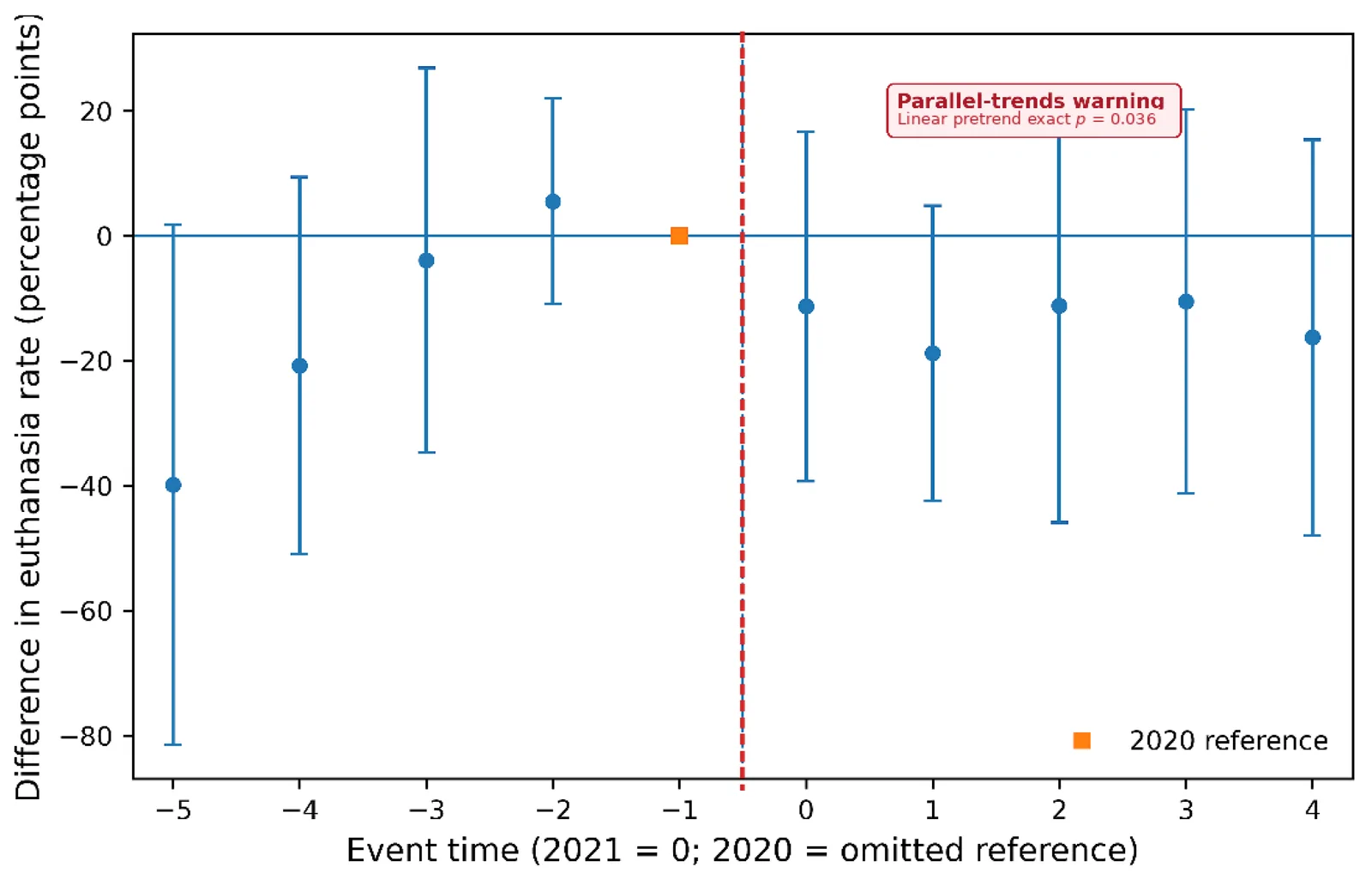
Event-study estimates for euthanasia rate. The 2020 coefficient is the omitted reference. Points show conversion-minus-contracted differences relative to 2020, and error bars show municipality-clustered t-based 95% confidence intervals. The dashed line separates the pre- and post-2021 periods.

Event-study diagnostics did not support a credible parallel-trend assumption for adoption or euthanasia. Relative to 2020, the 2016 adoption lead was +30.20 percentage points (exact coefficient-specific *p* = 0.007), and the 2016 euthanasia lead was −39.86 percentage points (*p* = 0.039) (Figure 2 and Figure 3). Complementary differential linear pre-trend tests estimated −8.22 percentage points per year for adoption (exact *p* = 0.004) and +10.60 percentage points per year for euthanasia (exact *p* = 0.036) during 2016–2020. Although the omnibus lead tests were less conclusive, the magnitude and systematic direction of the pre-period differences indicate that the groups were not on comparable trajectories before the 2021 boundary. Post-2021 adoption and euthanasia coefficients therefore cannot be interpreted as causal conversion effects.

For complete-case mean LOS and RMST365, neither the omnibus exact lead test nor the complementary linear pre-trend test rejected equal pre-period trajectories (Table 5). Post-2021 estimates were directionally consistent with slower throughput but remained imprecise. Complete event-study coefficients are reported in Supplementary Table S4.

The control-pool sensitivity analysis further constrained substantive interpretation. Excluding five newly digitized contracted controls changed the adoption estimate from +4.65 to −1.86 percentage points and reduced the complete-case LOS estimate from +64.93 to +31.67 days; no outcome had an exact *p*-value below 0.05. Thus, the direction or magnitude of several estimates depended materially on which controls were included. Detailed estimates are provided in Supplementary Table S6; this sensitivity does not imply that the newly digitized records were erroneous.

### 3.6. Censor-Aware Shelter Throughput

The RMST365 analysis included 44,407 dogs admitted during 2016–2024 after excluding five negative date intervals. In the primary panel, conversion was associated with +13.33 days of restricted mean time without a recorded disposition (cluster-t 95% CI, −37.42 to +64.09; exact *p* = 0.596) and +0.16 percentage points in the probability of no recorded disposition by day 365 (−7.16 to +7.49; *p* = 0.975) (Table 6). Excluding the newly digitized controls yielded +11.77 days (*p* = 0.657) and −3.08 percentage points (*p* = 0.457). The data were therefore consistent with the possibility of some delay during the first year but did not show a precise difference in the proportion unresolved at one year.

**Figure 4:**
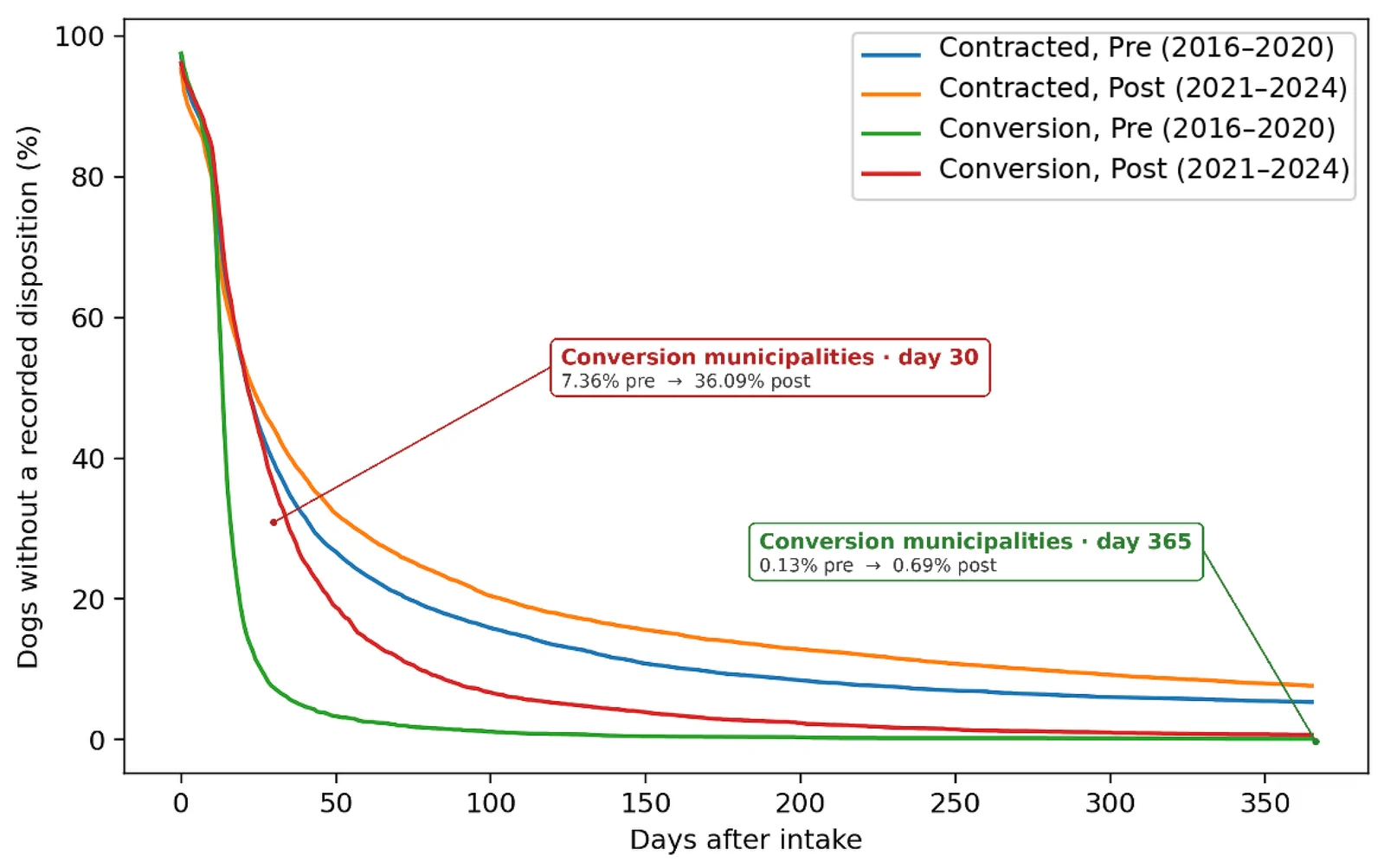
Dog-weighted probability of remaining without a recorded disposition through 365 days, by analytic group and period. Curves include 2016–2024 intake cohorts and are descriptive, unadjusted estimates rather than covariate-adjusted difference-in-differences estimates. Pre-period = 2016–2020; post-period = 2021–2024.

Descriptive curves showed that the visible change was concentrated early after intake. In conversion municipalities, the share without a recorded disposition on day 30 increased from 7.36% before 2021 to 36.09% afterward, whereas the day-365 share increased only from 0.13% to 0.69%. In contracted municipalities, the corresponding day-30 values were 39.32% and 44.16%, and the day-365 values were 5.35% and 7.67% (Figure 4). An exploratory dog-weighted resolution-time Cox estimate is reported in Supplementary Table S8 but was not used to define the primary conclusion.

### 3.7. Secondary Dog-Level Adoption and Euthanasia Pathways

**Figure 5:**
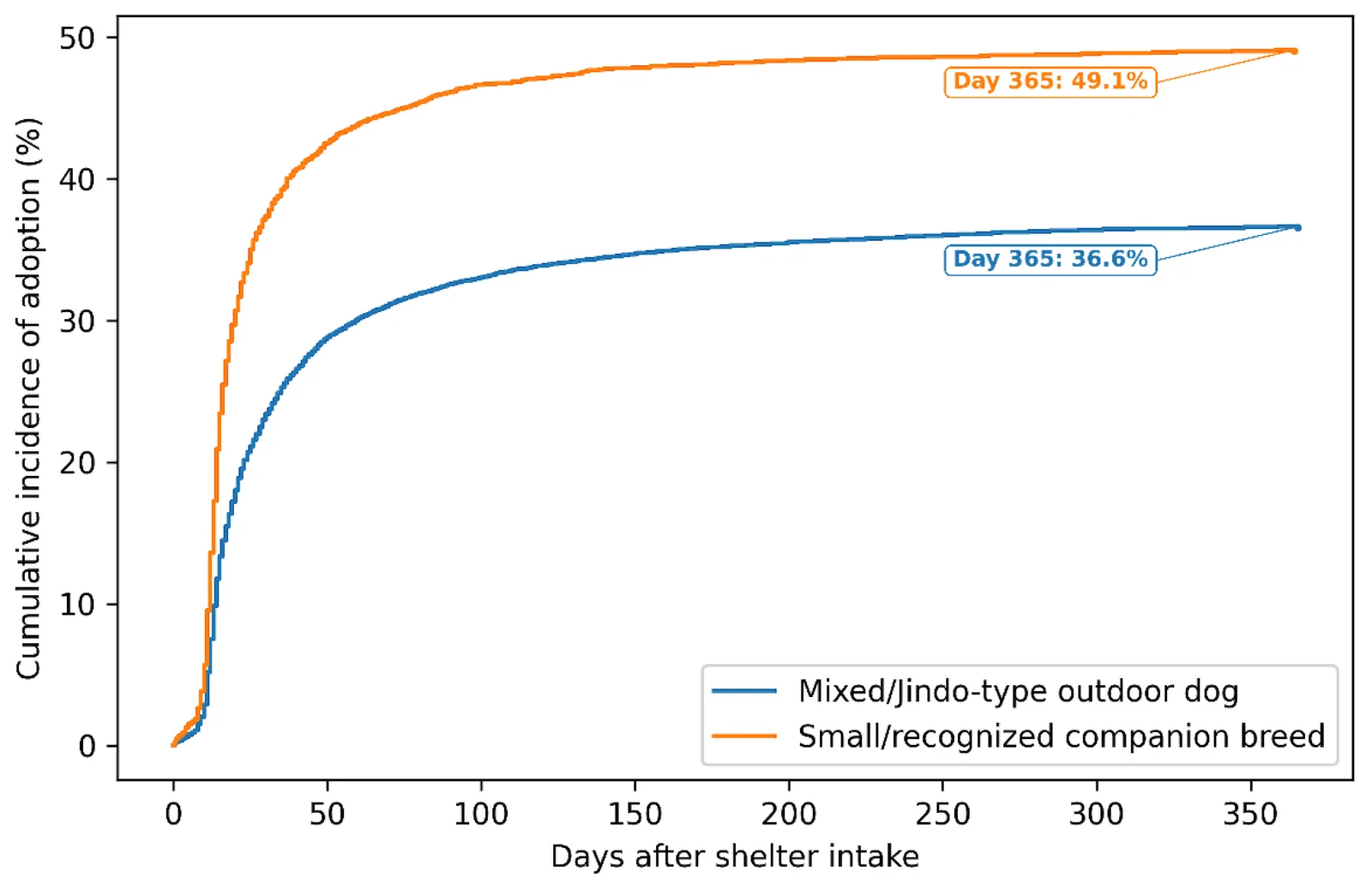
Cumulative incidence of adoption by dog phenotype in the harmonized dog-level survival subframe. Curves were estimated using the Aalen–Johansen estimator. Euthanasia, recorded death in care, return-to-owner, and other dispositions were treated as competing events.

**Figure 6:**
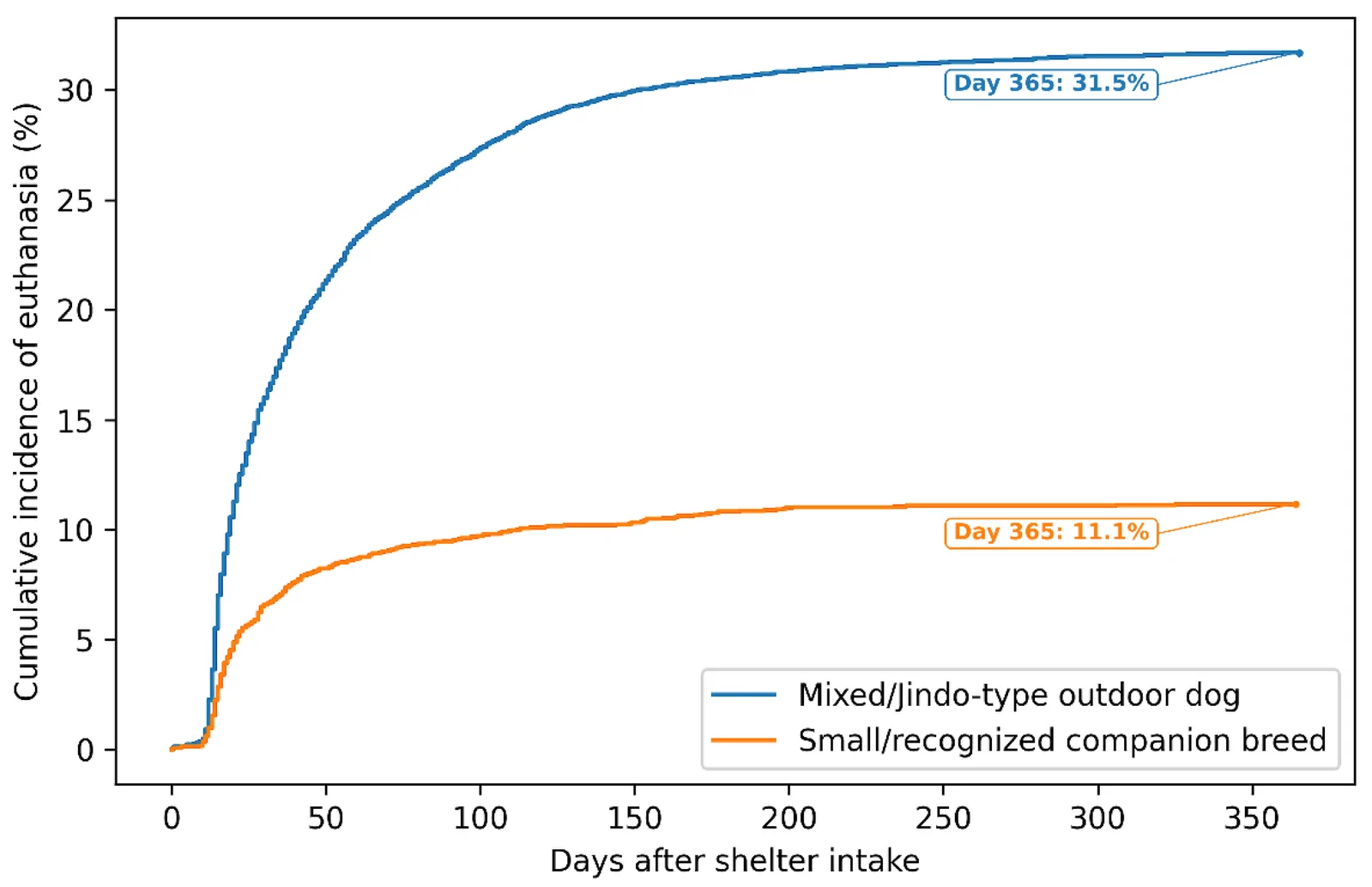
Cumulative incidence of euthanasia by dog phenotype in the harmonized dog-level survival subframe. Curves were estimated using the Aalen–Johansen estimator. Adoption, recorded death in care, return-to-owner, and other dispositions were treated as competing events.

The secondary harmonized survival subframe included 35,366 dogs from 12 municipalities. During the first year after intake, small or recognized companion breeds were adopted sooner and more often than mixed/Jindo-type outdoor dogs (Figure 5), whereas mixed/Jindo-type outdoor dogs were euthanized more often (Figure 6). These cumulative-incidence curves describe dog-level pathway differences before covariate adjustment and do not estimate the municipal conversion effect.

After adjustment for municipality and intake-year fixed effects, mixed/Jindo-type outdoor dogs had a lower adoption hazard (HR, 0.39; 95% CI, 0.35–0.45) and a higher euthanasia hazard (HR, 1.61; 95% CI, 1.37–1.90) than small or recognized companion breeds (Table 7). Puppies had a higher adoption hazard than adults, whereas senior and clinically compromised/injured dogs had lower adoption hazards. These estimates are secondary dog-level associations within the harmonized subframe and should not be used to infer a municipality-level conversion effect.

## 4. Discussion

This study addressed one primary policy question: whether shelter outcomes changed differently in municipalities that converted from contracted to direct operation than in municipalities that remained contracted. The findings did not support a uniform or causally interpretable conversion effect. Primary annual estimates were imprecise, adoption and euthanasia trajectories differed before 2021, and several point estimates changed with the comparison pool. The secondary dog-level analysis showed a more consistent dog-level welfare pattern: mixed/Jindo-type outdoor dogs and clinically compromised/injured dogs experienced less favorable placement pathways. Together, these findings support treating direct operation as a heterogeneous implementation package rather than a standardized intervention.

Direct evidence comparing public, private, and nonprofit shelter governance remains limited and context-dependent. A Portuguese survey found differences in funding, volunteer dependence, and operational vulnerability between municipal and association shelters without demonstrating a simple ownership advantage [34]. A U.S. case study reported early gains after privatization but later partnership failure when contractual obligations, accountability, goal alignment, communication, and governance were weak [35]. Public-administration scholarship also emphasizes that government shelters may serve open-admission or provider-of-last-resort mandates that differ from those of private or nonprofit shelters [36]. Accordingly, governance comparisons should measure contract terms, public service obligations, financing, accountability, and implementation content rather than rely on ownership labels alone.

For municipal management, conversion should trigger explicit documentation of what changed and when. Core performance indicators should include veterinary coverage, intake triage, isolation and treatment capacity, housing and population limits, staffing capacity, owner tracing, foster and transfer pathways, adoption promotion, long-stay review, euthanasia decision procedures, and record-closure completeness [20,21,22]. Contracted shelters require clear service specifications, reporting duties, welfare standards, and accountability mechanisms; directly operated shelters require comparable reporting of budget, staffing, clinical capacity, and service volume. Monitoring these operational components would make reform more actionable and allow municipalities to distinguish a change in administrative responsibility from a change in animal-care delivery.

The secondary dog-level findings identify populations that may require targeted welfare intervention. Mixed/Jindo-type outdoor dogs had a 61% lower adoption hazard and a 61% higher euthanasia hazard than small or recognized companion breeds, while clinically compromised/injured dogs had lower adoption hazards. General adoption promotion may therefore be insufficient if it does not address phenotype-related adopter preferences, medical needs, and barriers to placement. Earlier behavioral and medical assessment, individualized profiles, temporary fostering, socialization and handling support, inter-regional transfer, and targeted adoption outreach may improve opportunities for these dogs [14,17,18,20]. Senior and medically compromised animals may additionally benefit from prompt diagnosis, treatment planning, recovery foster care, and transparent communication of care needs to potential adopters.

Shelter throughput also has a direct welfare interpretation. The RMST365 and day-365 results did not show a precisely estimated persistent unresolved burden, while descriptive curves suggested that the main difference occurred during early processing. A longer early interval may reflect extended care, delayed administrative entry, congestion, transfer preparation, or a change in record-closing practice; these mechanisms cannot be distinguished in the current ledgers. Municipal dashboards should therefore report the proportions without a recorded disposition at 30, 90, and 365 days, distinguish medical, behavioral, transfer, and adoption-pending statuses, and require structured review of long-stay cases [13,20,21,30,31,37]. Standardized closure rules are necessary so that longer care intended to improve welfare is not conflated with administrative delay or unrecognized overcrowding.

Methodological constraints remain important but are not the scientific contribution of the study. Only four municipalities converted at the common boundary, conversion was observational, exact label permutation could not remove selection into conversion, adoption and euthanasia failed the pre-trend diagnostics, and estimates were sensitive to the control pool. Data systems also differed in unresolved-status and disposition-date conventions. These features limit the analysis to descriptive implementation evidence and prevent causal attribution of post-2021 differences to direct operation alone [22,23,25,26,27,28].

Additional limitations include the one-year horizon of RMST365, the inability to identify the reason for delayed disposition, retrospective phenotype and health recoding, and estimation of the secondary Cox models in a 12-municipality subframe. The study covered one Korean province and may not represent metropolitan, fully private, or differently regulated shelter systems. Future evaluations should prospectively define the components and timing of municipal reform, harmonize intake and disposition records before implementation, collect staffing and service-capacity measures, and include more conversion events across multiple regions. Such designs would better distinguish governance structure from the operational mechanisms that directly affect animal welfare.

## 5. Conclusions

The primary municipality-level analysis did not show a consistent improvement or deterioration in shelter outcomes after conversion to direct operation. Because the groups followed different pre-conversion trajectories and estimates changed with the comparison pool, the administrative label alone should not be interpreted as a causal intervention. The secondary dog-level analysis identified mixed/Jindo-type outdoor dogs and clinically compromised/injured dogs as groups with less favorable placement pathways. Regardless of governance structure, municipalities should evaluate standardized indicators of veterinary access, housing and population capacity, adoption, foster and transfer support, long-stay review, euthanasia decision processes, and record completeness, while directing additional clinical and placement resources to dogs at elevated risk.

## Figures and Tables

**Table 1:** Analytic groups and data contributions, 2016–2025.

Analytic Group	Municipalities, *n*	Dog Records, *n* (%)	Municipality–Years, *n*	Role in Analysis
Continuously direct	6	11,335 (16.7)	60	Descriptive reference; four municipalities also entered the harmonized survival subframe.
Continuously contracted	9	33,871 (49.8)	90	Primary DID controls; four municipalities entered the harmonized survival subframe.
2021-boundary conversion	4	15,624 (23.0)	40	Primary DID exposure group; all four entered the harmonized survival subframe.
Context/sensitivity	3	7202 (10.6)	30	Analyzed separately due to transition timing or implementation differences.
All municipalities	22	68,032 (100.0)	220	Province-wide dog-only census.

Note. DID = difference-in-differences. Continuously direct-operation municipalities served as descriptive references. The three context/sensitivity municipalities were excluded from the primary 2021-boundary DID analysis due to transition timing or implementation differences. Municipality-level classifications, data provenance, and implementation histories are provided in Supplementary Table S1. Percentages may not sum to 100.0 because of rounding.

**Table 2:** Dog-level case mix by analytic group in the 22-municipality census, *n* (%).

Characteristic	Category	Continuously Direct	Contracted	Conversion	Context/Sensitivity	All Municipalities
Breed/phenotype	Mixed/Jindo-type outdoor dog	9844 (86.8)	26,949 (79.6)	13,647 (87.3)	5097 (70.8)	55,537 (81.6)
Breed/phenotype	Small/recognized companion breed	1490 (13.1)	6922 (20.4)	1977 (12.7)	2105 (29.2)	12,494 (18.4)
Age group	Puppy/juvenile (<1 year)	5537 (48.8)	16,698 (49.3)	8155 (52.2)	2572 (35.7)	32,962 (48.5)
Age group	Adult (1–6 years)	5341 (47.1)	15,787 (46.6)	6958 (44.5)	4207 (58.4)	32,293 (47.5)
Age group	Senior (≥7 years)	451 (4.0)	1352 (4.0)	486 (3.1)	422 (5.9)	2711 (4.0)
Sex	Female	5805 (51.2)	17,462 (51.6)	8255 (52.8)	3529 (49.0)	35,051 (51.5)
Intake condition	Clinically compromised/injured	710 (6.3)	3921 (11.6)	1369 (8.8)	528 (7.3)	6528 (9.6)

Note. Percentages were calculated within each analytic group. Mixed/Jindo-type outdoor dogs included mixed-breed, Jindo/Jindo-type, Balbari, and related local outdoor-dog descriptors. Rare values omitted from the table included unknown breed (*n* = 1), explicitly unspecified age (*n* = 15), missing/unclassifiable age (*n* = 51), unknown health status (*n* = 1), and missing/unclassifiable health status (*n* = 3). Male sex and healthy/good intake condition were complementary categories. Neuter and microchip status were not analyzed because recording was inconsistent.

**Table 3:** Crude disposition and length-of-stay outcomes by analytic group, 2016–2025.

Group	Dogs, *n*	Adoption, %	Euthanasia, %	Death in Care, %	RTO, %	Other/Transfer, %	Unresolved/in Care or Unknown, %	Mean LOS, Days
Continuously direct	11,335	29.2	30.1	30.8	8.9	0.5	0.5	76.6
Contracted	33,871	43.2	24.2	17.7	11.0	0.8	3.0	74.1
Conversion	15,624	36.9	39.6	14.4	8.0	0.9	0.2	29.4
Context/sensitivity	7202	34.9	26.3	21.1	15.4	0.9	1.3	82.2
All municipalities	68,032	38.6	28.9	19.5	10.4	0.8	1.8	64.9

Note. Percentages were calculated within analytic groups. RTO = return-to-owner; LOS = length of stay. Mean LOS was calculated only for records with a valid disposition date. Unresolved/in-care and unresolved/unknown records were retained in disposition denominators; mean LOS and unresolved status should therefore be interpreted jointly. Recorded death in care denotes a death documented in the shelter ledger and does not imply death due to old age or a clinically adjudicated natural cause.

**Table 4:** Primary municipality–year difference-in-differences estimates with municipality-clustered confidence intervals and exact label-permutation *p*-values.

Outcome	Estimate (Cluster-t 95% CI)	Exact Permutation *p*-Value
Return-to-owner rate, percentage points	−2.19 (−7.05 to +2.67)	0.341
Adoption rate, percentage points	+4.65 (−16.98 to +26.28)	0.632
Recorded death-in-care rate, percentage points	+2.36 (−22.90 to +27.62)	0.846
Euthanasia rate, percentage points	−1.80 (−39.56 to +35.96)	0.922
Mean LOS among valid dispositions, days	+64.93 (−53.46 to +183.32)	0.234
Unresolved/in-care or unknown share, percentage points	−2.57 (−5.52 to +0.39)	0.095

Note. Models included municipality and year fixed effects. LOS = length of stay. Confidence intervals used municipality-clustered standard errors with finite-sample correction, a t reference, and 12 degrees of freedom. Exact two-sided *p*-values enumerated all 715 assignments of four conversion labels among 13 municipalities. Because conversion was observational, the permutation analysis is an exchangeability-based sensitivity diagnostic rather than design-based randomization inference.

**Table 5:** Pre-trend diagnostics for the primary 13-municipality panel.

Outcome	Differential Linear Pre-Trend	Exact *p*-Value	Omnibus Lead Exact *p*-Value
Adoption rate	−8.22 pp/year	0.004	0.172
Euthanasia rate	+10.60 pp/year	0.036	0.145
Complete-case mean LOS	+20.98 days/year	0.916	0.477
RMST365	−2.42 days/year	0.842	0.622

Note. Linear pre-trend models interacted conversion status with a centered year trend during 2016–2020. Omnibus tests jointly assessed the four event-study leads for 2016–2019 relative to 2020. Exact *p*-values used all 715 municipality-label assignments. pp = percentage points; LOS = length of stay; RMST365 = restricted mean time without a recorded disposition through 365 days.

**Table 6:** Censor-aware municipality–year difference-in-differences estimates through 365 days.

Analysis	RMST365, Days (95% CI; Exact *p*)	No Disposition by Day 365, Percentage Points (95% CI; Exact *p*)
Primary 13-municipality panel	+13.33 (−37.42 to +64.09); *p* = 0.596	+0.16 (−7.16 to +7.49); *p* = 0.975
Excluding newly digitized controls	+11.77 (−56.37 to +79.91); *p* = 0.657	−3.08 (−12.59 to +6.43); *p* = 0.457

Note. RMST365 = restricted mean time without a recorded disposition through 365 days. RMST365 is the mean of min (time to recorded disposition, 365 days). Dogs with no recorded disposition or a disposition after day 365 contributed 365 days. Analyses used 2016–2024 intake cohorts. Confidence intervals used municipality-clustered t references; exact *p*-values enumerated municipality-label assignments.

**Table 7:** Secondary cause-specific Cox proportional hazards models for adoption and euthanasia in the harmonized dog-level survival subframe.

Predictor	Reference	Adoption HR, 95% CI	Adoption *p*-Value	Euthanasia HR, 95% CI	Euthanasia *p*-Value
Mixed/Jindo-type outdoor dog	Small/recognized companion breed	0.39 (0.35–0.45)	<0.001	1.61 (1.37–1.90)	<0.001
Puppy/juvenile (<1 year)	Adult (1–6 years)	1.61 (1.40–1.85)	<0.001	0.81 (0.57–1.15)	0.238
Senior (≥7 years)	Adult (1–6 years)	0.78 (0.63–0.98)	0.031	1.27 (0.97–1.67)	0.081
Age unspecified	Adult (1–6 years)	1.67 (0.63–4.43)	0.305	1.45 (0.64–3.28)	0.372
Female	Male	0.93 (0.88–0.98)	0.006	0.97 (0.86–1.08)	0.550
Clinically compromised/injured	Healthy/good	0.70 (0.63–0.77)	<0.001	1.18 (0.97–1.44)	0.098

Note. Models included municipality and intake-year fixed effects and municipality-clustered standard errors. HR = hazard ratio; CI = confidence interval. The analysis included 35,366 dogs from 12 municipalities. The age-unspecified estimate was based on 15 dogs and was imprecise. Estimates describe dog-level associations within the harmonized subframe and are not the primary municipality-level governance estimand.

## Data Availability

The data presented in this study, including cleaned analytic outputs and analytic diagnostics, are available in the article and its Supplementary Materials, including Supplementary File S1 (Analysis_Workbook.xlsx). Executable analysis scripts are available from the corresponding author upon reasonable request. The underlying row-level municipal shelter ledgers were accessed under municipality-specific administrative permissions; they are not owned by the author and cannot be redistributed by the author. Requests for row-level access must be directed to and approved by the relevant municipalities, subject to their applicable data-use, administrative, and privacy conditions.

## Supplementary Materials

The following supporting information can be downloaded at: https://www.mdpi.com/article/10.3390/ani16162505/s1, Table S1: Municipality-level source coverage, implementation context, and analytic roles in the 22-municipality dog-only census, 2016–2025; Table S2: Dog-level case mix by analytic group in the 22-municipality census, n (%); Table S3: Detailed primary municipality-level difference-in-differences estimates with HC1, municipality-clustered, and exact permutation inference; Table S4: Complete event-study coefficients for diagnostic analyses, with 2020 as the omitted reference; Table S5: Linear and omnibus pretrend diagnostics for the primary municipal comparison; Table S6: Primary DID sensitivity analysis excluding five newly digitized contracted-control ledgers; Table S7: Primary censor-aware RMST365 and day-365 unresolved difference-in-differences estimates; Table S8: Exploratory resolution-time Cox sensitivity analysis; Table S9: Municipal date and disposition audit for the primary 13-municipality difference-in-differences panel; Table S10: Municipality-specific overall and common-calendar pre/post disposition and complete-case length-of-stay summaries, 2016–2025; Figure S1: Crude disposition distributions by analytic group (2016–2025); Figure S2: Annual dog intake volume in the primary 13-municipality DID panel (2016–2025); Figure S3: Analytic hierarchy and relation of primary, diagnostic, and secondary analyses; Supplementary File S1 (Analysis_Workbook.xlsx): machine-readable cleaned analytic outputs and analytic diagnostics, including complete event-study coefficients, HC1 robust standard error comparisons, exact permutation results, control-pool sensitivity estimates, censor-aware summaries, and exploratory resolution-time Cox regression outputs. Executable analysis scripts are available from the corresponding author upon reasonable request.
