# Supplementary material for: Beyond the Administrative Label: Municipal Shelter Conversion and Dog Outcomes in Korea, 2016–2025
Source: Animals (Basel). 2026 Aug 12;16(16):2505. doi: 10.3390/ani16162505 (PMC13508886; doi:10.3390/ani16162505)
Supplement: Supplementary file 1 [file animals-16-02505-s001.zip › Supplementary_Materials.pdf]

Supplementary Materials

Beyond the Administrative Label: Municipal Shelter Conversion and Dog Outcomes in Korea, 2016–2025

Byeong-Cheol Song

Agriculture and Livestock Division, Yeongyang County Office, Republic of Korea

This supplementary package contains Tables S1–S10, Figures S1–S3, and Supplementary File S1 (Analysis_Workbook.xlsx), which support the analytic hierarchy. Tables S3–S8 provide detailed diagnostic and sensitivity analyses for the primary municipality-level comparison, whereas the harmonized dog-level pathway analysis is secondary and exploratory. Supplementary File S1 contains machine-readable cleaned analytic outputs and analytic diagnostics, including complete event-study coefficients, HC1 robust standard error comparisons, exact permutation results, control-pool sensitivity estimates, censor-aware summaries, and exploratory resolution-time Cox regression outputs. Executable analysis scripts are available from the corresponding author upon reasonable request; use of the restricted municipal ledgers remains subject to municipal data-use approval. The underlying municipal ledgers are not reproduced in the public supplement.

Supplementary Methods

Analytic hierarchy and scope. The province-wide census included 68,032 dog records from 22 municipalities during 2016–2025. The primary scientific question was whether shelter outcomes changed differently in four municipalities that converted from contracted to direct operation than in nine continuously contracted controls; the primary municipality–year DID panel included 49,495 dogs and 130 municipality–years. Event-study, exact label-permutation, control-pool, and censor-aware analyses were diagnostic or sensitivity analyses of this same municipal estimand, not separate research objectives. The secondary, exploratory dog-level analysis used a harmonized 35,366-dog subframe from 12 municipalities to characterize adoption and euthanasia pathways by phenotype, age, sex, and intake health. Continuously direct municipalities served as descriptive references, while Yeongyang County, Gumi City, and Gimcheon City were retained as separate contextual or sensitivity cases (Figure S3).

Municipality-specific descriptive summaries. For Supplementary Table S10, outcome counts were aggregated within each municipality for 2016–2025 overall and for the common calendar periods 2016–2020 and 2021–2025. Outcome percentages use municipality-period dog intake as the denominator. Mean LOS is summarized from records with a valid nonnegative disposition interval and is presented together with the unresolved share. For Yeongyang County, Gumi City, and Gimcheon City, the 2021 split is a common calendar comparison and is not their municipality-specific conversion boundary.

Primary small-cluster inference. The primary 13-municipality DID included municipality and year fixed effects. HC1 heteroskedasticity-robust results are reported for comparison, but the principal uncertainty summaries use CR1 municipality-clustered standard errors, a t reference with $G - 1$ degrees of freedom, and exact studentized label-permutation sensitivity. All $C(13, 4) = 715$ assignments of four conversion labels among 13 municipalities were enumerated while retaining the common 2021 boundary and observed outcome paths. Because conversion was observational, exact permutation p-values are interpreted as exchangeability-based robustness diagnostics rather than design-based randomization probabilities.

Primary diagnostic analyses. Event-study models replaced the single DID term with conversion-by-year interactions and used 2020 as the omitted reference. The supplement reports coefficient-specific cluster-t confidence intervals, HC1 standard errors, coefficient-specific exact studentized permutation p-values, a four-lead omnibus diagnostic for 2016–2019, and a complementary differential linear-pretrend test over 2016–2020. A comparison-pool sensitivity analysis excluded five newly digitized contracted controls. These analyses evaluate the credibility and robustness of the primary municipal comparison and are not independent study objectives.

Primary censor-aware throughput analysis. The RMST365 analysis used 2016–2024 intake cohorts so that all dogs had 365 days of potential follow-up by 31 December 2025. RMST365 was defined as the mean of min(time to recorded disposition, 365 days). Dogs with no recorded disposition or with disposition after day 365 contributed 365 days and were classified as having no recorded disposition by day 365. Five negative rescue-to-disposition intervals were excluded from LOS, RMST, and resolution-time Cox analyses but retained in disposition-rate denominators.

Resolution-time sensitivity and secondary dog-level pathway analysis. The exploratory dog-level Cox model of time to any recorded disposition used administrative censoring on 31 December 2025, municipality and intake-year fixed effects, and municipality-clustered standard errors. It is retained only as a throughput sensitivity analysis because it is dog-weighted, assumes proportional hazards, and relies on few municipality clusters. Separately, the harmonized secondary survival subframe included 35,366 dogs from Yeongju, Yeongdeok, Yecheon, Bonghwa, Andong, Sangju, Seongju, Chilgok, Gyeongju, Uljin, Cheongdo, and Cheongsong. Nonparametric cumulative incidence functions for adoption and euthanasia used the Aalen–Johansen estimator with shelter intake as time zero. Cause-specific Cox models included phenotype, age, sex, intake health status, municipality fixed effects, and intake-year fixed effects, with municipality-clustered standard errors. These dog-level models characterize adoption and euthanasia pathways and do not estimate the municipal conversion effect.

Table S1. Municipality-level source coverage, implementation context, and analytic roles in the 22-municipality dog-only census, 2016–2025.

Municipality	Analytic group	Dog records, n	Share of census, %	Coverage	Municipality-years	Ledger provenance	Implementation context and analytic role
Yeongju City	Continuously direct	3,375	5.0	2016–2025	10	Original/previously available	Direct operation throughout 2016–2025; descriptive reference; included in the harmonized survival subframe.
Yeongdeok County	Continuously direct	2,344	3.4	2016–2025	10	Original/previously available	Direct operation throughout 2016–2025; descriptive reference; included in the harmonized survival subframe.
Yecheon County	Continuously direct	2,132	3.1	2016–2025	10	Original/previously available	Direct operation throughout 2016–2025; descriptive reference; included in the harmonized survival subframe.
Bonghwa County	Continuously direct	413	0.6	2016–2025	10	Original/previously available	Direct operation throughout 2016–2025; descriptive reference; included in the harmonized survival subframe.
Yeongcheon City	Continuously direct	2,922	4.3	2016–2025	10	Newly digitized in revision	Direct operation throughout 2016–2025; newly digitized descriptive reference.
Ulleung County	Continuously direct	149	0.2	2016–2025	10	Newly digitized in revision	Direct operation throughout 2016–2025; low-intake island descriptive reference; newly digitized.
Andong City	Continuously contracted	1,681	2.5	2016–2025	10	Original/previously available	Continuously contracted; primary DID control; retained in exclusion sensitivity and harmonized survival subframe.
Sangju City	Continuously contracted	3,781	5.6	2016–2025	10	Original/previously available	Continuously contracted; primary DID control; retained in exclusion sensitivity and harmonized survival subframe.
Seongju County	Continuously contracted	3,149	4.6	2016–2025	10	Original/previously available	Continuously contracted; primary DID control; retained in exclusion sensitivity and harmonized survival subframe.
Chilgok County	Continuously contracted	2,867	4.2	2016–2025	10	Original/previously available	Continuously contracted; primary DID control; retained in exclusion sensitivity and harmonized survival subframe.
Pohang City	Continuously contracted	10,021	14.7	2016–2025	10	Newly digitized in revision	Continuously contracted; newly digitized primary DID control; excluded in provenance sensitivity.
Gyeongsan City	Continuously contracted	5,654	8.3	2016–2025	10	Newly digitized in revision	Continuously contracted; newly digitized primary DID control; excluded in provenance sensitivity.
Uiseong County	Continuously contracted	1,792	2.6	2016–2025	10	Newly digitized in revision	Continuously contracted; newly digitized primary DID control; excluded in provenance sensitivity.
Mungyeong City	Continuously contracted	2,978	4.4	2016–2025	10	Newly digitized in revision	Continuously contracted; newly digitized primary DID control; excluded in provenance sensitivity.
Goryeong County	Continuously contracted	1,948	2.9	2016–2025	10	Newly digitized in revision	Continuously contracted; newly digitized primary DID control; excluded in provenance sensitivity.

Municipality	Analytic group	Dog records, n	Share of census, %	Coverage	Municipality-years	Ledger provenance	Implementation context and analytic role
Gyeongju City	Conversion	8,096	11.9	2016–2025	10	Original/previously available	Primary conversion municipality; direct-operation conversion with a new shelter opening in 2021; survival subframe.
Uljin County	Conversion	3,250	4.8	2016–2025	10	Original/previously available	Primary conversion municipality; new 50-dog shelter opened in January 2021; survival subframe.
Cheongdo County	Conversion	2,974	4.4	2016–2025	10	Original/previously available	Primary conversion municipality; staged direct operation with formal center opening in 2024; survival subframe.
Cheongsong County	Conversion	1,304	1.9	2016–2025	10	Original/previously available	Primary conversion municipality; non-construction transition with limited staffing and external veterinary referral; survival subframe.
Yeongyang County	Independent sensitivity case	571	0.8	2016–2025	10	Original/previously available	Independent 2023 sensitivity case; excluded from primary DID because timing and operational conditions differed.
Gumi City	Late-conversion context	3,507	5.2	2016–2025	10	Original/previously available	Late-conversion contextual case; excluded from primary DID because timing and veterinary-care arrangements differed.
Gimcheon City	Late-conversion context	3,124	4.6	2016–2025	10	Original/previously available	Late-conversion contextual case; excluded from primary DID because timing and veterinary-care arrangements differed.
Total	All analytic groups	68,032	100.0	2016–2025	220	Seven newly digitized ledgers	**Province-wide dog-only census; seven newly digitized ledgers contributed 25,464 dogs.**

Note. Seven newly digitized ledgers (Yeongcheon, Ulleung, Pohang, Gyeongsan, Uiseong, Mungyeong, and Goryeong) contributed 25,464 dogs (37.4% of the census). Five were continuously contracted controls and contributed 22,393 dogs to the primary 13-municipality difference-in-differences (DID) analysis. Gunwi County was excluded after its incorporation into Daegu Metropolitan City in July 2023. Analytic denominators were restricted to verified dog records. Implementation descriptions summarize the classifications used in the manuscript; detailed local administrative records remain subject to municipal data-use restrictions.

Table S2. Dog-level case mix by analytic group in the 22-municipality census, n (%).

Characteristic	Category	Continuously direct	Contracted	Conversion	Context/sensitivity	All municipalities
Breed/phenotype	Mixed/Jindo-type outdoor dog	9,844 (86.8)	26,949 (79.6)	13,647 (87.3)	5,097 (70.8)	55,537 (81.6)
Breed/phenotype	Small/recognized companion breed	1,490 (13.1)	6,922 (20.4)	1,977 (12.7)	2,105 (29.2)	12,494 (18.4)
Breed/phenotype	Unknown breed	1 (0.0)	0 (0.0)	0 (0.0)	0 (0.0)	1 (0.0)
Age group	Puppy/juvenile (<1 yr)	5,537 (48.8)	16,698 (49.3)	8,155 (52.2)	2,572 (35.7)	32,962 (48.5)
Age group	Adult (1–6 yr)	5,341 (47.1)	15,787 (46.6)	6,958 (44.5)	4,207 (58.4)	32,293 (47.5)
Age group	Senior (≥7 yr)	451 (4.0)	1,352 (4.0)	486 (3.1)	422 (5.9)	2,711 (4.0)
Age group	Age unspecified	0 (0.0)	14 (0.0)	0 (0.0)	1 (0.0)	15 (0.0)
Age group	Missing/unclassifiable	6 (0.1)	20 (0.1)	25 (0.2)	0 (0.0)	51 (0.1)
Sex	Female	5,805 (51.2)	17,462 (51.6)	8,255 (52.8)	3,529 (49.0)	35,051 (51.5)
Sex	Male	5,530 (48.8)	16,409 (48.4)	7,369 (47.2)	3,673 (51.0)	32,981 (48.5)
Intake condition	Clinically compromised/injured	710 (6.3)	3,921 (11.6)	1,369 (8.8)	528 (7.3)	6,528 (9.6)

Characteristic	Category	Continuously direct	Contracted	Conversion	Context/sensitivity	All municipalities
Intake condition	Healthy/good	10,622 (93.7)	29,949 (88.4)	14,255 (91.2)	6,674 (92.7)	61,500 (90.4)
Intake condition	Unknown health status	1 (0.0)	0 (0.0)	0 (0.0)	0 (0.0)	1 (0.0)
Intake condition	Missing/unclassifiable	2 (0.0)	1 (0.0)	0 (0.0)	0 (0.0)	3 (0.0)

Note. Percentages were calculated within analytic groups. Mixed/Jindo-type outdoor dogs included mixed-breed, Jindo/Jindo-type, Balbari, and related local outdoor-dog descriptors. Fifteen records were explicitly coded as age unspecified; 51 additional age descriptors and three intake-condition entries were missing or unclassifiable. Neuter and microchip status were not analyzed because recording was inconsistent.

Table S3. Detailed primary municipality-level difference-in-differences estimates with HC1, municipality-clustered, and exact permutation inference.

Outcome	Unit	DID estimate	HC1 SE	HC1 p	CR1 clustered SE	Cluster-t p	Cluster-t 95% CI	Exact studentized p	Exact coefficient p
Return-to-owner rate	percentage points	−2.19	1.35	0.105	2.23	0.346	−7.05 to 2.67	0.341	0.269
Adoption rate	percentage points	4.65	4.94	0.347	9.93	0.648	−16.98 to 26.28	0.632	0.614
Recorded death-in-care rate	percentage points	2.36	5.23	0.651	11.59	0.842	−22.90 to 27.62	0.846	0.857
Euthanasia rate	percentage points	−1.80	7.63	0.814	17.33	0.919	−39.56 to 35.96	0.922	0.895
Mean LOS among records with valid disposition date	days	64.93	38.77	0.094	54.34	0.255	−53.46 to 183.32	0.234	0.434
Unresolved/in-care or unknown share	percentage points	−2.57	1.58	0.104	1.36	0.083	−5.52 to 0.39	0.095	0.218

Note. Models included municipality and year fixed effects and compared four conversion municipalities with nine continuously contracted controls (13 municipalities, 130 municipality-years, and 49,495 dogs). DID = difference-in-differences; HC1 = heteroskedasticity-consistent variance estimator; CR1 = cluster-robust variance estimator with finite-sample correction; LOS = length of stay. Cluster-t confidence intervals used 12 degrees of freedom. Exact two-sided p-values enumerated all 715 assignments of four conversion labels; the studentized exact p-value was the primary permutation diagnostic.

Table S4. Complete event-study coefficients for diagnostic analyses, with 2020 as the omitted reference.

Panel A. Adoption rate (percentage points)

Year	Event time	Coefficient	Clustered SE	Cluster-t p	Cluster-t 95% CI	HC1 SE	Exact studentized p
2016	−5	30.20	10.66	0.015	6.98 to 53.43	9.08	0.007
2017	−4	24.20	8.48	0.014	5.73 to 42.67	8.60	0.013
2018	−3	8.90	7.21	0.241	−6.82 to 24.61	11.37	0.236
2019	−2	2.42	5.28	0.654	−9.08 to 13.93	9.14	0.674
2020	−1	0.00 (reference)	—	—	—	—	—
2021	0	13.70	10.61	0.221	−9.43 to 36.83	8.91	0.204
2022	1	19.10	9.75	0.074	−2.15 to 40.35	7.63	0.070
2023	2	16.26	11.23	0.173	−8.21 to 40.74	8.19	0.143
2024	3	13.42	10.47	0.224	−9.39 to 36.23	8.45	0.214
2025	4	26.48	11.55	0.041	1.32 to 51.64	9.23	0.022

Panel B. Euthanasia rate (percentage points)

Year	Event time	Coefficient	Clustered SE	Cluster-t p	Cluster-t 95% CI	HC1 SE	Exact studentized p
2016	−5	−39.86	19.12	0.059	−81.52 to 1.80	17.93	0.039
2017	−4	−20.78	13.85	0.159	−50.95 to 9.39	14.25	0.130
2018	−3	−3.94	14.14	0.785	−34.74 to 26.85	15.50	0.778
2019	−2	5.53	7.53	0.477	−10.88 to 21.94	11.84	0.459
2020	−1	0.00 (reference)	—	—	—	—	—
2021	0	−11.30	12.85	0.396	−39.29 to 16.69	10.68	0.415
2022	1	−18.78	10.85	0.109	−42.41 to 4.86	9.24	0.120
2023	2	−11.17	15.94	0.497	−45.90 to 23.57	13.55	0.491
2024	3	−10.51	14.11	0.471	−41.26 to 20.24	11.99	0.449
2025	4	−16.29	14.54	0.285	−47.97 to 15.40	12.06	0.267

Table S4 (continued). Complete event-study coefficients for diagnostic analyses, with 2020 as the omitted reference.

Panel C. Complete-case mean LOS (days)

Year	Event time	Coefficient	Clustered SE	Cluster-t p	Cluster-t 95% CI	HC1 SE	Exact studentized p
2016	−5	−35.32	126.03	0.784	−309.92 to 239.27	94.92	0.905
2017	−4	−102.76	194.97	0.608	−527.56 to 322.03	154.47	0.917
2018	−3	56.18	38.50	0.170	−27.71 to 140.07	57.48	0.180
2019	−2	36.40	42.28	0.406	−55.72 to 128.52	61.31	0.586
2020	−1	0.00 (reference)	—	—	—	—	—
2021	0	31.17	26.28	0.259	−26.09 to 88.44	56.50	0.260
2022	1	64.80	38.20	0.116	−18.44 to 148.04	59.17	0.108
2023	2	57.83	41.27	0.186	−32.09 to 147.75	53.89	0.211
2024	3	54.19	40.42	0.205	−33.89 to 142.26	52.50	0.256
2025	4	71.16	41.10	0.109	−18.39 to 160.71	56.06	0.123

Panel D. RMST365 (days; 2016–2024 intake cohorts)

Year	Event time	Coefficient	Clustered SE	Cluster-t p	Cluster-t 95% CI	HC1 SE	Exact studentized p
2016	−5	20.46	46.41	0.667	−80.65 to 121.58	37.75	0.676
2017	−4	1.22	46.43	0.980	−99.95 to 102.39	36.09	0.989
2018	−3	25.62	18.71	0.196	−15.14 to 66.39	22.93	0.208
2019	−2	17.92	20.67	0.403	−27.12 to 62.95	24.95	0.564
2020	−1	0.00 (reference)	—	—	—	—	—
2021	0	11.11	16.25	0.507	−24.30 to 46.51	24.21	0.494
2022	1	40.54	27.01	0.159	−18.31 to 99.39	26.22	0.138
2023	2	32.54	27.84	0.265	−28.11 to 93.20	24.43	0.253
2024	3	21.32	24.71	0.405	−32.51 to 75.15	22.60	0.403

Note. Event time 0 corresponds to 2021, and event time -1 (calendar year 2020) is the omitted reference. Coefficients are conversion-minus-contracted differences relative to 2020. Cluster-t confidence intervals used municipality-clustered standard errors and 12 degrees of freedom. Exact p-values used the absolute coefficient-specific municipality-clustered studentized statistic across all 715 label assignments. LOS = length of stay; RMST365 = restricted mean time without a recorded disposition through 365 days.

Table S5. Linear and omnibus pretrend diagnostics for the primary municipal comparison.

Panel A. Primary 13-municipality panel: differential linear pretrend and four-lead omnibus diagnostics

Outcome	Slope unit	Linear slope	Clustered SE	Cluster-t p	Exact linear p	Omnibus W	Omnibus F	Asymptotic chi-square p	Exact omnibus p
Adoption rate	percentage points/year	−8.22	2.79	0.012	0.004	12.55	3.14	0.014	0.172
Euthanasia rate	percentage points/year	10.60	5.53	0.079	0.036	12.66	3.16	0.013	0.145
Mean LOS among valid dispositions	days/year	20.98	44.02	0.642	0.916	5.03	1.26	0.284	0.477
RMST365 censor-aware	days/year	−2.42	12.73	0.852	0.842	3.88	0.97	0.423	0.622

Panel B. Eight-municipality original-control sensitivity: differential linear pretrend

Outcome	Slope unit	Linear slope	Clustered SE	Cluster-t p	Exact linear p	Cluster df	Observations	Assignments
Adoption rate	percentage points/year	−6.17	2.69	0.056	0.057	7	40	70
Euthanasia rate	percentage points/year	9.24	6.20	0.180	0.143	7	40	70
Mean LOS among valid dispositions	days/year	−29.55	14.05	0.074	0.114	7	40	70
RMST365 censor-aware	days/year	−18.84	11.84	0.155	0.114	7	40	70

Note. Linear-pretrend models interacted conversion status with a centered year trend during 2016–2020. The primary-panel omnibus test jointly assessed the four 2016–2019 event-study leads relative to 2020. Exact omnibus p-values used the same test statistic across all 715 municipality-label assignments. The asymptotic chi-square p-value is shown for comparison and was not used as the primary few-cluster diagnostic. LOS = length of stay; RMST365 = restricted mean time without a recorded disposition through 365 days.

Table S6. Primary DID sensitivity analysis excluding five newly digitized contracted-control ledgers.

Outcome	Unit	Primary-panel estimate	Exclusion estimate	Change	Exclusion HC1 p	Exclusion CR1 SE	Exclusion cluster-t p	Exclusion 95% CI	Exact p
Return-to-owner rate	percentage points	−2.19	−2.36	−0.17	0.169	2.61	0.396	−8.53 to 3.81	0.457
Adoption rate	percentage points	4.65	−1.86	−6.50	0.754	14.16	0.899	−35.35 to 31.64	0.857
Recorded death-in-care rate	percentage points	2.36	14.13	11.76	0.007	11.42	0.256	−12.89 to 41.14	0.257
Euthanasia rate	percentage points	−1.80	−4.96	−3.16	0.526	18.25	0.794	−48.11 to 38.20	0.771
Mean LOS among records with valid disposition date	days	64.93	31.67	−33.27	0.155	35.29	0.399	−51.79 to 115.12	0.400
Unresolved/in-care or unknown share	percentage points	−2.57	−4.80	−2.24	0.130	2.41	0.086	−10.50 to 0.89	0.086

Note. The exclusion sensitivity retained four conversion municipalities and four original contracted controls (Andong, Sangju, Seongju, and Chilgok), yielding eight municipalities, 80 municipality–years, 27,102 dogs, and all C(8, 4) = 70 label assignments. DID = difference-in-differences; HC1 = heteroskedasticity-consistent variance estimator; CR1 = cluster-robust variance estimator with finite-sample correction; LOS = length of stay. Excluded controls were Pohang, Gyeongsan, Uiseong, Mungyeong, and Goryeong. Change equals the exclusion estimate minus the primary-panel estimate. This analysis evaluated comparison-pool and provenance sensitivity and does not imply that newly digitized ledgers were invalid.

Table S7. Primary censor-aware RMST365 and day-365 unresolved difference-in-differences estimates.

Analysis	Outcome	Unit	Estimate	HC1 SE	HC1 p	CR1 SE	Cluster-t p	Cluster-t 95% CI	Exact p	Assignments
Primary 13-municipality panel	Restricted mean time without recorded disposition through 365 days	days	13.33	13.07	0.308	23.30	0.578	−37.42 to 64.09	0.596	715

Analysis	Outcome	Unit	Estimate	HC1 SE	HC1 p	CR1 SE	Cluster-t p	Cluster-t 95% CI	Exact p	Assignments
8-municipality original-control	Restricted mean time without recorded disposition through 365 days	days	11.77	17.13	0.492	28.82	0.695	−56.37 to 79.91	0.657	70
Primary 13-municipality panel	Probability of no recorded disposition by 365 days	percentage points	0.16	3.20	0.960	3.36	0.963	−7.16 to 7.49	0.975	715
8-municipality original-control	Probability of no recorded disposition by 365 days	percentage points	−3.08	4.10	0.452	4.02	0.469	−12.59 to 6.43	0.457	70

Note. DID = difference-in-differences; RMST365 = restricted mean time without a recorded disposition through 365 days. RMST365 is the mean of min(time to recorded disposition, 365 days). Dogs with no recorded disposition or a disposition after day 365 contributed 365 days. Analyses used 2016–2024 intake cohorts. Primary-panel estimates used 13 municipalities and 117 municipality–year observations; exclusion estimates used eight municipalities and 72 municipality–year observations. Exact p-values are studentized label-permutation diagnostics.

Table S8. Exploratory resolution-time Cox sensitivity analysis.

Analysis	Dogs, n	Recorded dispositions, n	Censored, n	Invalid dates excluded	Clusters	HR	Cluster-t 95% CI	Cluster-t p	Asymptotic p
Primary 13-municipality panel	49,490	47,893	1,597	5	13	0.63	0.44 to 0.90	0.016	0.005
Excluding newly digitized controls	27,098	25,984	1,114	4	8	0.54	0.35 to 0.82	0.010	<0.001

Note. The outcome was time to any recorded disposition, with administrative censoring on 31 December 2025. Models included municipality and intake-year fixed effects and municipality-clustered standard errors. HR = hazard ratio. HR < 1 indicates a lower recorded-disposition hazard after conversion relative to the difference-in-differences comparison. These dog-weighted estimates differ from the unweighted municipality-year RMST365 estimand, assume proportional hazards, and remain vulnerable to few-cluster inference; they are exploratory.

Table S9. Municipal date and disposition audit for the primary 13-municipality difference-in-differences panel.

Panel A. Date completeness and impossible negative intervals

Municipality	Analytic group	Dog records	Valid disposition dates	Missing dates	Negative intervals	Valid-date coverage, %
Gyeongju	Conversion	8,096	8,093	0	3	100.0
Uljin	Conversion	3,250	3,232	18	0	99.4
Cheongdo	Conversion	2,974	2,974	0	0	100.0
Cheongsong	Conversion	1,304	1,299	5	0	99.6
Andong	Original contracted control	1,681	1,159	522	0	68.9
Sangju	Original contracted control	3,781	3,676	104	1	97.2
Seongju	Original contracted control	3,149	2,963	186	0	94.1
Chilgok	Original contracted control	2,867	2,866	1	0	100.0
Pohang	Newly digitized contracted control	10,021	9,955	66	0	99.3
Gyeongsan	Newly digitized contracted control	5,654	5,536	118	0	97.9
Uiseong	Newly digitized contracted control	1,792	1,789	3	0	99.8

Municipality	Analytic group	Dog records	Valid disposition dates	Missing dates	Negative intervals	Valid-date coverage, %
Mungyeong	Newly digitized contracted control	2,978	2,978	0	0	100.0
Goryeong	Newly digitized contracted control	1,948	1,945	2	1	99.8

Panel B. Disposition-category counts

Municipality	Return-to-owner	Adoption	Recorded death in care	Euthanasia	Other/transfer	Unresolved/in care	Unresolved/unknown
Gyeongju	930	3,076	1,065	2,966	59	0	0
Uljin	112	1,622	158	1,294	45	0	19
Cheongdo	166	921	809	1,052	26	0	0
Cheongsong	46	146	223	876	8	0	5
Andong	285	821	39	11	3	522	0
Sangju	346	1,708	972	536	111	108	0
Seongju	137	1,331	719	748	9	0	205
Chilgok	184	2,039	417	218	8	1	0
Pohang	1,543	4,470	1,352	2,572	15	69	0
Gyeongsan	690	2,242	1,326	1,265	12	119	0
Uiseong	80	278	485	935	9	5	0
Mungyeong	379	1,436	357	801	5	0	0
Goryeong	80	316	330	1,116	104	2	0

Table S9 (continued). Records with a disposition date preceding the rescue date.

Panel C. Negative rescue-to-disposition intervals

Municipality	Source row	Rescue date	Disposition date	Interval, days
Gyeongju	1083	2018-04-11	2018-04-07	−4
Gyeongju	1816	2018-10-10	2018-10-08	−2
Gyeongju	6332	2022-05-30	2022-05-29	−1
Sangju	36	2017-08-10	2016-09-23	−321
Goryeong	122	2017-09-05	2017-09-02	−3

Note. Disposition categories were harmonized as return-to-owner, adoption, recorded death in care, euthanasia, other/transfer, unresolved/in care, and unresolved/unknown. Recorded death in care denotes the administrative category recorded in the ledger and does not imply old age or a clinically adjudicated natural cause. The five negative intervals were retained in disposition-rate denominators but excluded from length-of-stay (LOS), RMST365, and resolution-time Cox analyses.

Table S10. Municipality-specific overall and common-calendar pre/post disposition and complete-case length-of-stay summaries, 2016–2025.

Panel A. Continuously direct municipalities

Municipality	Period	Dogs, n	Adoption, %	Euthanasia, %	Recorded death in care, %	RTO, %	Unresolved, %	Mean LOS, days
	**Overall**	**3,375**	**26.2**	**16.6**	**42.9**	**12.0**	**1.5**	**113.1**
**Yeongju City**	Pre	1,642	30.0	1.4	55.9	12.4	0.0	137.8
	Post	1,733	22.7	31.0	30.5	11.7	2.9	88.9
	**Overall**	**2,344**	**17.3**	**43.8**	**31.5**	**7.2**	**0.0**	**44.9**
**Yeongdeok County**	Pre	842	17.8	36.0	39.2	7.0	0.0	52.0
	Post	1,502	17.0	48.2	27.2	7.3	0.1	40.9
	**Overall**	**2,132**	**33.2**	**43.1**	**15.8**	**7.6**	**0.0**	**61.0**
**Yecheon County**	Pre	729	25.4	49.0	19.2	6.3	0.0	56.6
	Post	1,403	37.3	40.1	14.0	8.2	0.0	63.2
	**Overall**	**413**	**32.2**	**50.6**	**9.9**	**6.3**	**1.0**	**47.6**
**Bonghwa County**	Pre	121	27.3	62.0	2.5	8.3	0.0	35.6
	Post	292	34.2	45.9	13.0	5.5	1.4	52.7
	**Overall**	**2,922**	**38.8**	**19.0**	**31.3**	**8.2**	**2.2**	**75.9**
**Yeongcheon City**	Pre	1,029	55.8	5.0	27.6	10.6	0.0	80.6
	Post	1,893	29.5	26.6	33.4	7.0	3.3	73.3
	**Overall**	**149**	**18.1**	**67.1**	**4.7**	**7.4**	**2.0**	**60.4**
**Ulleung County**	Pre	124	16.9	72.6	5.6	4.0	0.0	67.4
	Post	25	24.0	40.0	0.0	24.0	12.0	20.9

Table S10 (continued). Municipality-specific overall and common-calendar pre/post summaries.

Panel B. Continuously contracted municipalities

Municipality	Period	Dogs, n	Adoption, %	Euthanasia, %	Recorded death in care, %	RTO, %	Unresolved, %	Mean LOS, days
Andong City	**Overall**	**1,681**	**48.8**	**0.7**	**2.3**	**17.0**	**31.1**	**79.7**
	Pre	845	51.4	1.3	4.6	14.3	28.3	117.1
	Post	836	46.3	0.0	0.0	19.6	33.9	38.7
Sangju City	**Overall**	**3,781**	**45.2**	**14.2**	**25.7**	**9.2**	**2.9**	**106.5**
	Pre	2,047	28.3	22.5	39.8	8.2	0.5	113.0
	Post	1,734	65.1	4.4	9.1	10.3	5.6	98.3
Seongju County	**Overall**	**3,149**	**42.3**	**23.8**	**22.8**	**4.4**	**6.5**	**147.7**
	Pre	1,401	28.3	33.3	35.0	3.2	0.1	144.7
	Post	1,748	53.4	16.1	13.0	5.3	11.6	150.4
Chilgok County	**Overall**	**2,867**	**71.1**	**7.6**	**14.5**	**6.4**	**0.0**	**27.4**
	Pre	1,452	71.5	5.2	16.0	6.8	0.0	23.9
	Post	1,415	70.7	10.0	13.1	6.0	0.1	31.0
Pohang City	**Overall**	**10,021**	**44.1**	**25.4**	**13.5**	**15.4**	**1.5**	**55.9**
	Pre	5,329	40.1	24.9	19.4	15.6	0.0	61.8
	Post	4,692	48.6	26.0	6.7	15.1	3.2	48.9
Gyeongsan City	**Overall**	**5,654**	**39.0**	**21.0**	**23.0**	**12.2**	**4.5**	**114.6**
	Pre	2,339	51.0	12.6	25.9	10.4	0.0	118.4
	Post	3,315	30.6	26.9	21.0	13.5	7.7	111.7
Uiseong County	**Overall**	**1,792**	**15.3**	**50.8**	**27.0**	**4.5**	**1.9**	**43.5**
	Pre	774	12.0	75.3	9.2	3.2	0.0	33.7
	Post	1,018	17.9	32.1	40.6	5.4	3.3	51.3
Mungyeong City	**Overall**	**2,978**	**48.1**	**26.7**	**11.9**	**12.7**	**0.4**	**21.8**
	Pre	1,401	48.7	31.8	7.1	12.2	0.0	19.7
	Post	1,577	47.6	22.2	16.2	13.2	0.7	23.6
Goryeong County	**Overall**	**1,948**	**15.9**	**56.5**	**16.4**	**4.1**	**1.7**	**34.1**
	Pre	856	13.0	75.4	7.2	4.4	0.0	17.5
	Post	1,092	18.1	41.8	23.6	3.8	3.1	47.6

Table S10 (continued). Municipality-specific overall and common-calendar pre/post summaries.

Panel C. 2021-boundary conversion municipalities

Municipality	Period	Dogs, n	Adoption, %	Euthanasia, %	Recorded death in care, %	RTO, %	Unresolved, %	Mean LOS, days
**Gyeongju City**	**Overall**	**8,096**	**38.0**	**36.6**	**13.2**	**11.5**	**0.0**	**22.6**
	Pre	3,373	35.0	35.9	18.8	10.1	0.0	16.2
	Post	4,723	40.2	37.2	9.1	12.4	0.0	27.2
**Uljin County**	**Overall**	**3,250**	**49.9**	**39.8**	**4.9**	**3.4**	**0.6**	**46.0**
	Pre	1,915	31.7	63.2	2.2	2.5	0.0	16.3
	Post	1,335	76.0	6.3	8.7	4.9	1.4	89.3
**Cheongdo County**	**Overall**	**2,974**	**31.0**	**35.4**	**27.2**	**5.6**	**0.0**	**25.0**
	Pre	1,252	20.5	53.4	17.4	8.5	0.0	16.3
	Post	1,722	38.6	22.3	34.3	3.5	0.0	31.3
**Cheongsong County**	**Overall**	**1,304**	**11.2**	**67.2**	**17.1**	**3.5**	**0.4**	**39.8**
	Pre	311	10.9	55.0	30.9	3.2	0.0	49.7
	Post	993	11.3	71.0	12.8	3.6	0.5	36.7

Table S10 (continued). Municipality-specific overall and common-calendar pre/post summaries.

Panel D. Independent sensitivity and late-conversion context municipalities

Municipality	Period	Dogs, n	Adoption, %	Euthanasia, %	Recorded death in care, %	RTO, %	Unresolved, %	Mean LOS, days
	**Overall**	**571**	**59.4**	**4.9**	**22.9**	**7.2**	**1.2**	**164.7**
**Yeongyang County**	Pre	275	68.4	8.4	18.5	4.4	0.0	168.2
	Post	296	51.0	1.7	27.0	9.8	2.4	161.4
	**Overall**	**3,507**	**31.5**	**29.9**	**18.7**	**17.9**	**1.2**	**56.9**
**Gumi City**	Pre	962	38.9	33.4	10.9	16.8	0.0	71.3
	Post	2,545	28.8	28.5	21.6	18.3	1.7	51.3
	**Overall**	**3,124**	**34.2**	**26.3**	**23.6**	**14.1**	**1.5**	**95.5**
**Gimcheon City**	Pre	1,327	45.4	21.1	22.3	10.9	0.0	109.3
	Post	1,797	26.0	30.1	24.5	16.4	2.6	85.0

Note. Values are unadjusted descriptive summaries. The preperiod was 2016–2020 and the postperiod was 2021–2025 for all municipalities. For Yeongyang County, Gumi City, and Gimcheon City, the 2021 split is a common-calendar comparison rather than the municipality-specific conversion boundary. Recorded death in care is the administrative ledger category and does not imply old age or a clinically adjudicated natural cause. Mean length of stay (LOS) was calculated from records with valid nonnegative disposition intervals and should be interpreted jointly with unresolved status. Unresolved combines in-care and unresolved/unknown records. RTO = return-to-owner.

Supplementary Figures

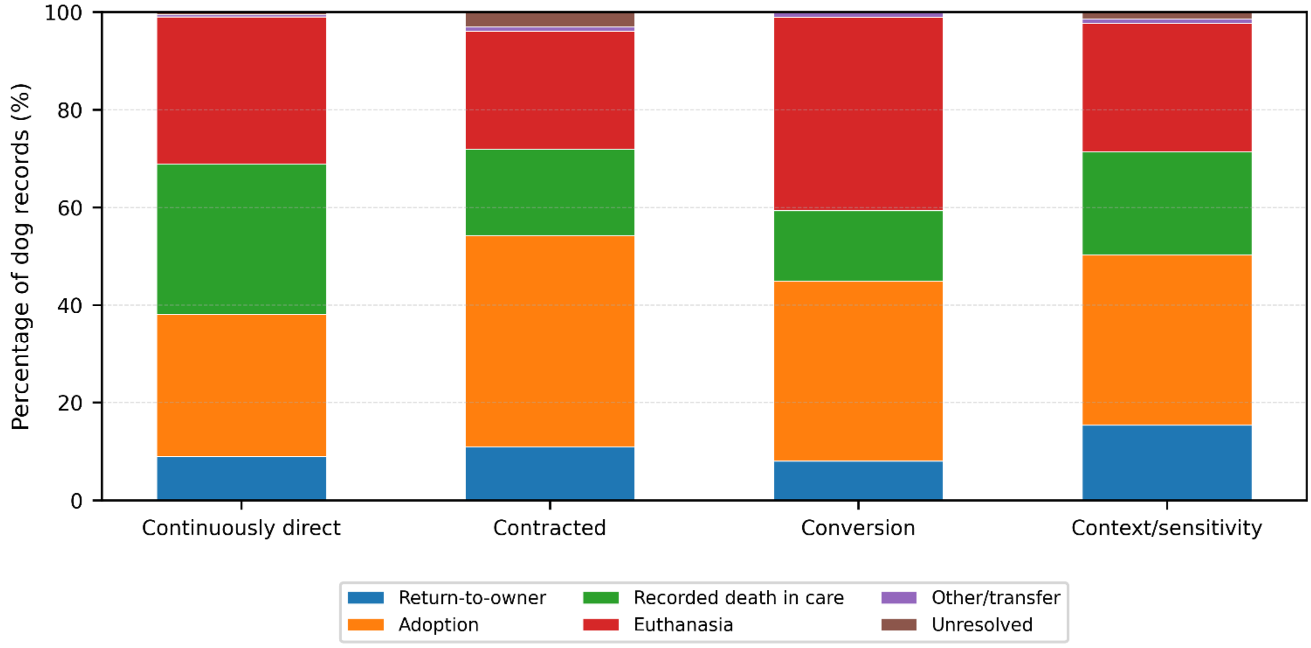

Figure S1. Crude disposition distributions by analytic group (2016-2025). Bars show percentages of 68,032 dog records from 22 municipalities classified as return-to-owner, adoption, recorded death in care, euthanasia, other/transfer, or unresolved/in care or unknown. In the unadjusted data, conversion municipalities had a higher euthanasia share than continuously contracted municipalities (39.6% vs 24.2%) and a shorter mean LOS (29.4 vs 74.1 days). These crude contrasts reflect case mix, operational practices, recording conventions, and governance status and should not be interpreted causally.

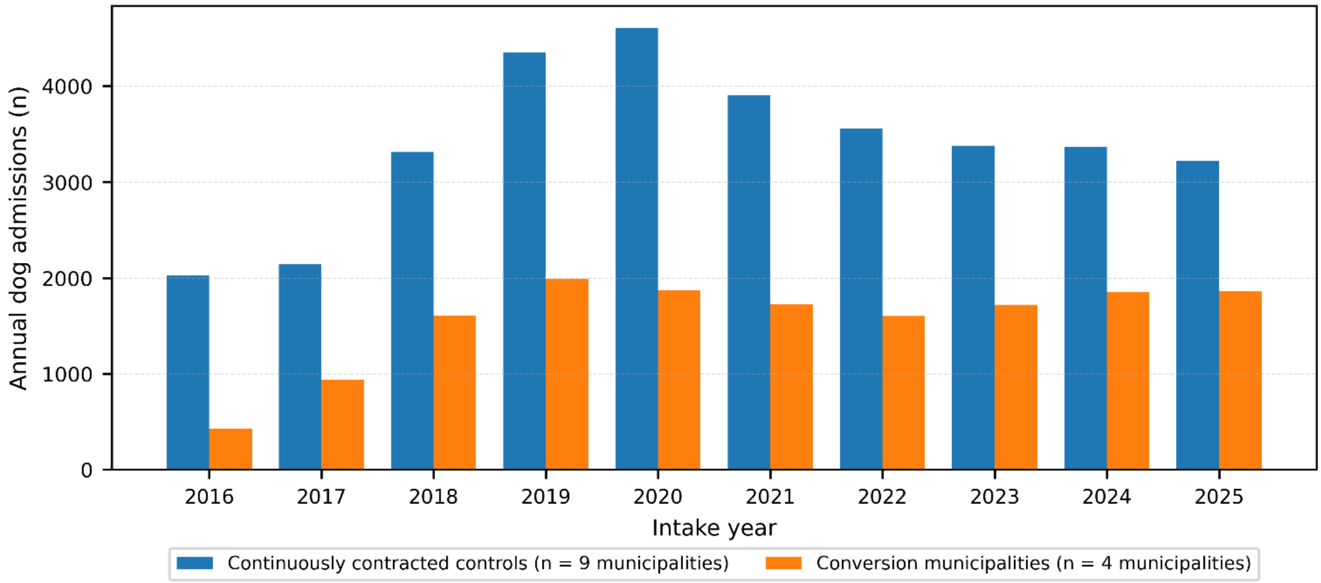

Figure S2. Annual dog intake volume in the primary 13-municipality DID panel (2016–2025). Grouped bars show annual admissions to nine continuously contracted controls and four conversion municipalities. Intake increased markedly in both groups from 2016 through 2020 and then stabilized after 2021. Counts are not population-standardized.

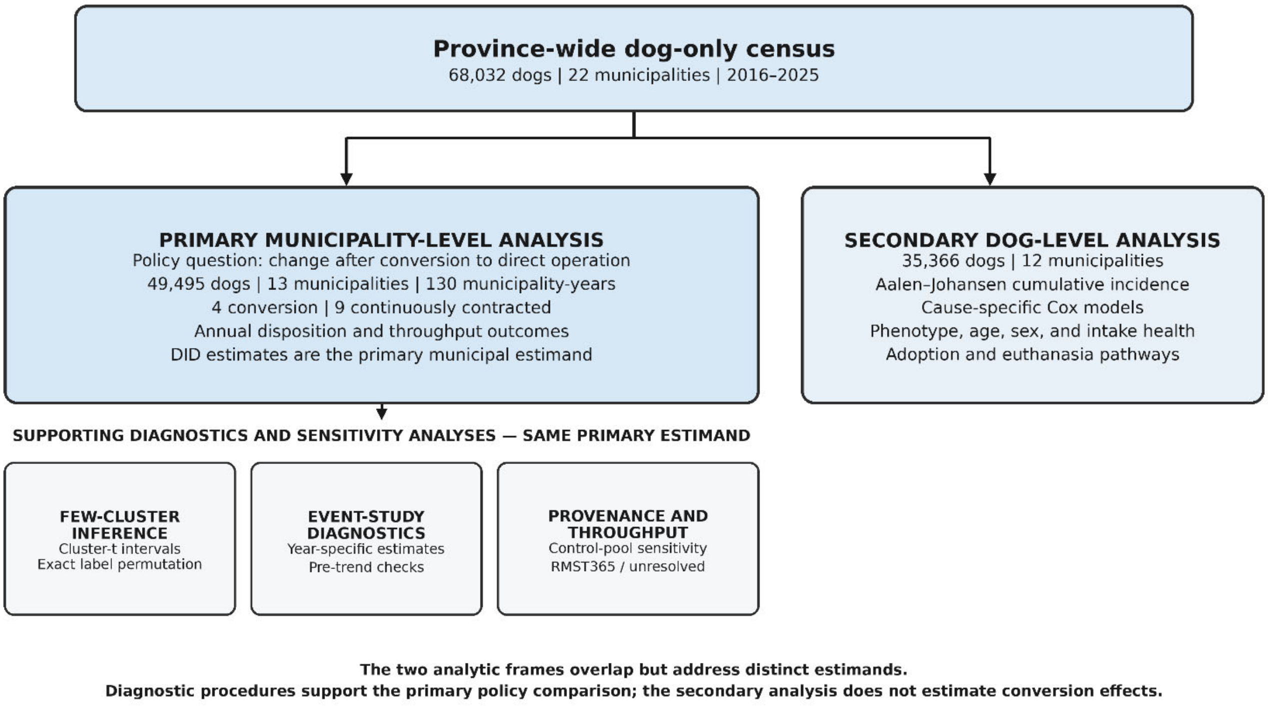

Figure S3. Analytic hierarchy and relation of primary, diagnostic, and secondary analyses. The province-wide dog-only census (68,032 dogs; 22 municipalities) generated two overlapping, non-sequential analytic frames. The primary municipality-level panel (49,495 dogs; 13 municipalities; 130 municipality–years) addressed the policy question of shelter conversion. Exact label-permutation inference, event-study and pretrend checks, comparison-pool sensitivity, and censor-aware throughput analyses supported that same primary estimand and were not separate research objectives. The secondary harmonized dog-level subframe (35,366 dogs; 12 municipalities) described adoption and euthanasia pathways by dog characteristics and did not estimate the municipal conversion effect.
